# Identification of Small Molecules Blocking the *Pseudomonas aeruginosa* Type III Secretion System Protein PcrV

**DOI:** 10.3390/biom11010055

**Published:** 2021-01-04

**Authors:** Charlotta Sundin, Michael Saleeb, Sara Spjut, Liena Qin, Mikael Elofsson

**Affiliations:** Department of Chemistry, Umeå University, SE-901 87 Umeå, Sweden; michael.saleeb@umu.se (M.S.); sara.spjut@umu.se (S.S.); lnqin@hotmail.com (L.Q.); mikael.elofsson@umu.se (M.E.)

**Keywords:** *Pseudomonas aeruginosa*, type III secretion system, PcrV, virulence inhibitors, screening, surface plasmon resonance, infection

## Abstract

*Pseudomonas aeruginosa* is an opportunistic bacterial pathogen that employs its type III secretion system (T3SS) during the acute phase of infection to translocate cytotoxins into the host cell cytoplasm to evade the immune system. The PcrV protein is located at the tip of the T3SS, facilitates the integration of pore-forming proteins into the eukaryotic cell membrane, and is required for translocation of cytotoxins into the host cell. In this study, we used surface plasmon resonance screening to identify small molecule binders of PcrV. A follow-up structure-activity relationship analysis resulted in PcrV binders that protect macrophages in a *P. aeruginosa* cell-based infection assay. Treatment of *P. aeruginosa* infections is challenging due to acquired, intrinsic, and adaptive resistance in addition to a broad arsenal of virulence systems such as the T3SS. Virulence blocking molecules targeting PcrV constitute valuable starting points for development of next generation antibacterials to treat infections caused by *P. aeruginosa*.

## 1. Introduction

Infectious diseases continue to constitute a threat to public health and a serious cause of morbidity and mortality worldwide. The alarming emergence rate of antimicrobial resistance is estimated to result in 10 million annual deaths with a cost of $100 trillion by 2050 [1]. The gram-negative bacterium *Pseudomonas aeruginosa* is an opportunistic pathogen and one of the most frequent causes of urinary tract, bloodstream, and burn wound infections as well as hospital-acquired pneumonia and cystic fibrosis-associated infections worldwide [2]. *P. aeruginosa* is a “superbug” with a unique capacity to develop resistance [3]. This is due to a combination of acquired, intrinsic, and adaptive resistance. Acquired resistance results from horizontal gene transfer and mutations leading to reduced uptake, efflux pump overexpression, target mutations, and expression of antibiotic modifying enzymes such as extended spectrum β-lactamases. The intrinsic resistance stems from a generally low outer membrane permeability, β-lactamase production, and constitutive expression of efflux pumps. Adaptive resistance is the result of triggering factors such as antibiotics, biocides, polyamines, pH, anaerobiosis, cations, and carbon sources as well as social behavior in biofilm formation. These factors modulate the expression of genes that lead to increased resistance. Taken together, these features have resulted in multi-drug-resistant *P. aeruginosa* strains for which no effective antibiotic treatment is available, and these strains are becoming more frequent [4]. The ability of *P. aeruginosa* to rapidly acquire or develop resistance against multiple classes of antimicrobials narrows down and complicates the selection of antimicrobial therapy, which is crucial in optimizing clinical outcome [2,5]. Furthermore, infections caused by antibiotic-resistant *P. aeruginosa* are associated with increased rates of morbidity and mortality, particularly in critically ill and immunocompromised individuals such as cystic fibrosis and cancer patients [2]. In such patients, approaches targeting virulence factors have the potential to prevent infection or to augment the effect of conventional antibiotics.

*P. aeruginosa* uses a broad arsenal of virulence factors, such as type IV pili, flagella, quorum sensing, biofilm formation, and different secretion systems (type I–IV and VI) to adapt to and survive within diverse harsh environmental settings and under minimal nutritional requirements [6,7,8,9,10]. Several of these systems, predominantly quorum sensing, biofilm formation, and type III and VI secretion systems, have been exploited in the search for novel therapeutic agents [11] A recent example is the report on tanshinones, a class of natural products blocking T3SS biogenesis in *P. aeruginosa* in vitro and in vivo [12].

**Figure 1:**
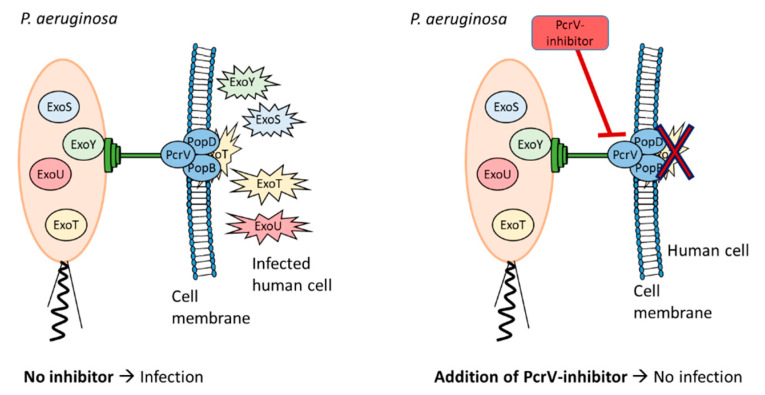
Schematic representation of the type III secretion system (T3SS): the T3SS has a syringe-like structure with two rings spanning the bacterial membranes and a needle protruding from the surface with the PcrV protein at its tip. Upon contact with the eukaryotic cell, the exoenzymes are translocated into the eukaryotic cell via the translocation-pore (PopB and PopD), which is dependent on the PcrV protein for its function. A PcrV inhibitor will block translocation, and no toxins will be transported into the eukaryotic cell.

Like several other gram-negative pathogens, *P. aeruginosa* utilizes its type III secretion system (T3SS) during the acute phase of infection to inject effector proteins (toxins) into the eukaryotic cytoplasm to subvert the host and to evade the immune defense [13]. To date, four known toxins, exoenzymes, secreted by *P. aeruginosa* T3S have been described: ExoU, a potent phospholipase; ExoS and ExoT, closely related bifunctional enzymes with ADP-ribosylation and GTPase activity; and ExoY, an adenylate cyclase [14,15]. The core of the T3SS is a nanosyringe structure that consists of a basal body spanning the bacterial membrane and an extracellular needle-like structure protruding from the bacterial surface and designed to deliver the effector proteins upon contact with a host cell (Figure 1) [16,17]. Although, the exact mechanism of the translocation remains poorly understood, it is believed that translocation of the effector protein is triggered by contact with the host cell. The PcrV protein forms an oligomeric ring structure, most likely a pentamer, at the tip of the needle that is crucial for a functional T3SS. [18,19] In addition, two hydrophobic translocator proteins, PopB and PopD [20,21,22], assemble with the PcrV protein at the tip of the T3SS needle and are inserted into the host cell membrane to form a functional pore (Figure 1) [23,24,25,26].

One approach to develop novel antibacterial agents is to block pathogenicity by inhibiting virulence instead of targeting bacterial viability [27]. Several authors suggest that a low probability for development of resistance is one of the key advantages of using virulence systems as targets for novel anti-infectives [28,29]. Resistance to compounds targeting virulence factors will most likely not evolve and spread in endogenous bacterial flora, since these bacteria do not express the specific virulence targets. The T3SS of *P. aeruginosa* and other gram-negative pathogens have been explored as targets for the development of virulence-blocking antibacterial agents [30,31]. A number of synthetic and natural compounds have been demonstrated to block the T3SS of *P. aeruginosa*, resulting in attenuation of infection in vitro and in vivo [32,33,34,35,36,37]. However, most of these compounds have been identified by phenotypic screening and the mode of action at the molecular level remains unknown.

PcrV is an attractive therapeutic target for the development of virulence blocking compounds against *P. aeruginosa*. Several studies have shown that PcrV is essential for a functional T3SS and intoxication of the host cell. Deletion of the *pcrV* gene (Δ*pcrV*) results in a mutant that is incapable of delivering the T3S toxins into the eukaryotic cells, thus abolishing in vitro and in vivo cytotoxicity [15,25,33,38]. In addition, it was found that active and passive immunization against PcrV greatly increase the survival rate in animal models of *P. aeruginosa*-induced lung infection [39]. Since PcrV is present on the exterior of the bacterium, the challenge of entry into the bacterial cell and the action of efflux pumps are circumvented. Importantly, the T3SS in *P. aeruginosa* is essential to block macrophage phagocytosis and the anti-PcrV antibody was demonstrated to successfully protect the macrophage from infection [35,39]. Later, many monoclonal and polyclonal anti-PcrV antibodies were shown to protect against *P. aeruginosa* infection in a variety of animal models [39,40,41,42,43] and anti-PcrV antibodies have been advanced to clinical trials [18,44,45]. Despite the essential role of PcrV in translocation mechanism, to date, there is no structural information, e.g., crystal structures available of the complete PcrV protein although a homology model has been built based on the crystal structure of LcrV from *Yersinia* [46], and to the best of our knowledge, small molecules blocking PcrV have not been described. Two recent studies describe surface plasmon resonance (SPR) screening against the analogues in *Shigella* (IpaD) and in *Salmonella* (SipD), resulting in low affinity binders; however, virulence blocking activity of these compounds have not been established [47,48].

Surface plasmon resonance (SPR) is a powerful and versatile biophysical technique for the detection of small molecule-biomolecule interactions, complementary with other techniques such as nuclear magnetic resonance (NMR) spectroscopy and X-ray crystallography [49]. SPR is sensitive and allows kinetic and thermodynamic evaluation, which makes it an excellent choice for the detection of low molecular weight analytes (e.g., 100–300 Da) and low-affinity interactions (i.e., high µM to mM). Moreover, one advantage that most SPR technologies offer nowadays is the availability of multiple biosensor channels, which allows the study of multiple proteins in parallel.

In this study, we describe SPR screening of a library of 7600 diverse small molecules to identify nontoxic PcrV binders with efficacy in a cell-based *P. aeruginosa* infection model at µM concentrations that can potentially be used as starting points for the development of a virulence blocking molecule.

## 2. Materials and Methods

### 2.1. Protein Expression and Purification

The PcrV gene (amino acid 20-294) was cloned into NcoI/Acc65I sites in the pETHis1a plasmid and transformed into the competent *E. coli* strain BL(DE3) (Novagen), generating a PcrV-protein lacking the first 19 amino acids and having a His-tag in the N-terminal. Three milliliters of overnight cultures of BL21(DE3) (pETHis1a, pcrV) grown in Luria Broth (LB) containing 50 µg/mL Kanamycin were added to 600 ml TYM-5052 medium, grown for 4 h; shook at 37 °C; and after the temperature was shifted to 26 °C, incubated overnight. The culture was then centrifuged, and pellet was washed in phosphate buffered saline (PBS) and then frozen for 24 h, after which lysis buffer was added and the sample was sonicated. Purification was carried out using gravity flow on Econo-Pac columns (Bio-Rad) packed with 2 mL of Ni^2+^ charged nitrilotriacetic acid (NTA) agarose (Qiagen) and equilibrated with lysis buffer. The column was washed with 10× the bead volume using wash buffer II, and the PcrV protein was eluted using 5× the bead volume of elution buffer. For all buffer ingredients, see the Supplementary Materials. The eluate was dialyzed in PBS, loaded on a 12% sodium dodecyl sulfate (SDS) gel, and stained using Coomassie. A very strong band was seen corresponding to PcrV, and the concentration was measured to 6.87 mg/mL. The purified PcrV protein was then used in SPR screening.

### 2.2. Surface Plasmon Resonance

The SPR screening was performed using the ProteOn^TM^ XPR36 Protein Interaction Array System (Bio-Rad Laboratories Inc., Hercules, CA, USA) with ProteOn GLH sensor chip (Bio-Rad Laboratories Inc., Hercules, CA, USA), and the results were analyzed with the ProteOn Manager software^TM^ (Bio-Rad Laboratories Inc., Hercules, CA, USA), Excel 2016 (Microsoft Office), and GraphPad Prism 7.04. All SPR experiments were performed at 25 °C. The multichannel module of the ProteOn system was in the vertical position during protein immobilization. The proteins were attached to the surface of a GLH sensor chip via amine coupling chemistry. A buffer scouting was performed to elucidate the optimal binding conditions of PcrV to the GLH sensor chip. First, channels 1–4 on the chip surface were activated using a 1:1 mixture of 40 mM 1-ethyl-3-(3-dimethylaminopropyl)carbodiimide hydrochloride (EDAC) (Bio-Rad Laboratories Inc., Hercules, CA, USA) and 10 mM *N*-hydroxysulfosuccinimide (sulfo-NHS, Bio-Rad Laboratories Inc., Hercules, CA, USA) for 300 s at 30 µL/min. Next, the proteins PcrV (33,398.6 Da, 6.37 mg/mL) was diluted in 10 mM NaOAc (pH 4, 4.5, and 5) to 0.05 mg/mL. PcrV was immobilized in channel 1–3 for 540 s at 30 µL/min. Channel 4 was flushed with 10 mM NaOAc (pH 5) for 540 s at 30 µL/min. As deactivating agent, a 1:1 mixture of 1 M ethanolamine and milli-Q filtered water was used. The deactivating solution was injected over all four channels for 300 s at 30 µL/min. Phosphate buffered saline with Tween (PBST; 10 mM phosphate buffer pH 7.4, 140 mM NaCl, 2.7 mM KCl, and 0.05% Tween 20) was used as the running buffer. The final amount of immobilized protein was measured in resonance units (RU) relative to the signal of the baseline prior activation. Sodium acetate of pH 5 and 4.5 gave comparable results (21,000 and 20,000 RU), while sodium acetate of pH 4 gave lower immobilization (16,000 RU) of PcrV to the surface. Immobilization of PcrV diluted in sodium acetate of pH 4.5 in one channel and of pH 5 in one channel were used in the beginning of the screening procedure (for plate 1–26). Since no difference was found between the two pH values, the PcrV protein was diluted in sodium acetate at pH 5 only (in two channels) from plate 27 and the rest of the screen. The immobilization procedure for the screening was performed as described above with the addition that the control protein carbonic anhydrase isoenzyme II from bovine erythrocytes (CAII, Sigma-Aldrich, article number 2522) was immobilized in one channel that was activated and deactivated as described above. CAII (1 mg/mL in PBS) was deactivated when diluted in 10 mM sodium acetate (pH 4) to 0.05 mg/mL. Immobilization level of the deactivated CAII was 18,000 RU. Deactivated CAII was used as a control protein according to the recommendations by the ProteOn manufacturer (Bio-Rad Laboratories Inc., Hercules, CA, USA). As running buffer, we employed PBS (pH 7.4, 0.05% Tween 20).

**Figure 2:**
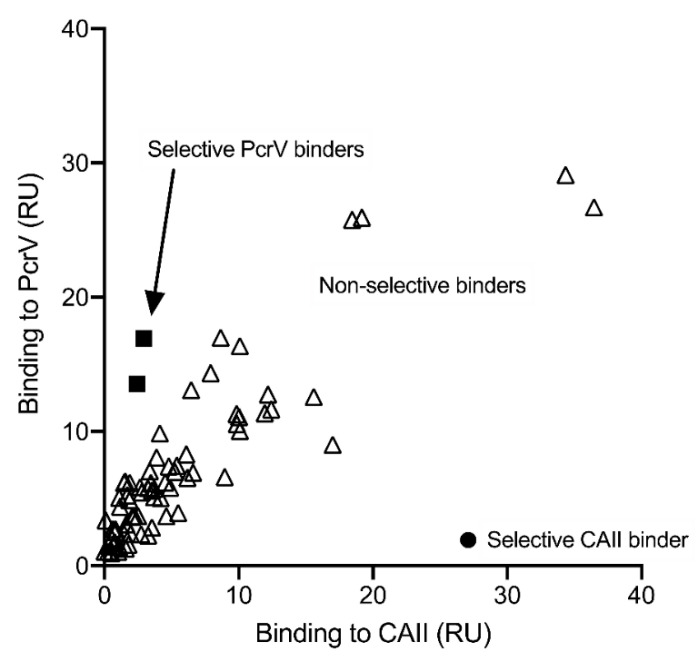
The *x*-axis shows the binding levels to the immobilized deactivated control protein arbonic anhydrase isoenzyme II from bovine erythrocytes (CAII), and the *y*-axis shows the binding levels (the average of two channels) to the immobilized PcrV protein for compounds from one exemplified screening plate. The binding levels (in resonance units (RU)) were blank subtracted, molecular weight adjusted, and multiplied by 100. The values on the *y*-axis show an average value of the binding levels to PcrV proteins immobilized in two channels while the binding level on the *x*-axis is from CAII immobilized in one channel. Two compounds that bind to PcrV with a higher selectivity than to CAII are shown as filled squares (“selective PcrV binders”, one is **H1** (Table 1)). One selective binder to CAII are marked with a filled circle (“selective CAII binder”). The remaining compounds are shown as triangles.

After ligand immobilization, the multichannel module of the ProteOn system was shifted to the horizontal position. The screening compound library consisted of 7600 compounds (95 plates and 80 compounds/plate) provided by the Laboratories for Chemical Biology Umeå (LCBU), Umeå University. The compounds were selected to cover the chemical space occupied of around 40,000 diverse compounds (Diverset, ChemBridge, San Diego, CA, USA). The compounds had a molecular weight between 250.17 and 449.97 g/mol and cLogP between −5.62 and 11.12 and, thus, contained fragments as well as lead-like and drug-like molecules. The analytes, i.e., the small molecule screening compounds, were prepared from DMSO stock solution (5 mM) in PBST buffer (0.05% Tween 20) to achieve a final concentration of 100 µM with a final DMSO concentration of 5% in 96-well plates (Proteon Standard Microplates, 98 wells, Bio-Rad Laboratories Inc., Hercules, CA, USA). The plates were sealed (ProteOn Microplate sealing film, Bio-Rad Laboratories Inc., Hercules, CA, USA) when the plate was prepared. The running buffer was PBST (0.05% Tween 20) containing 5% DMSO. The analytes were injected at a flow rate of 100 µL/min, contact time was 60 s, and dissociation time was 60–120 s. Solvent correction was performed continuously during screening to account for bulk response of DMSO. Each sensor chip was used for up to a week, allowing immobilization and then screening of 8–16 plates. The data was baseline-aligned, processed by channel-referencing with channel 4, and solvent-corrected. The data, i.e., the response, was then exported to Excel (Microsoft Office), where it was adjusted for molecular weight of the analyte (and multiplied with 100). Molecules with two times higher binding to PcrV compared to the control protein, CAII, were considered as selective binders, exemplified in Figure 2. Occasionally, air bubbles in the diluted sample wells interfered.

The analytes, i.e., the hits from the screening campaign that did not inhibit growth of *P. aeruginosa*, and synthesized analogs were further evaluated in dose-response experiments using SPR technique. First, the PcrV protein was immobilized in two channels as described above while keeping a blank channel which was activated and deactivated as described above. The multichannel module of the ProteOn system was in horizontal position during the small molecule interaction studies. They were serially diluted to (400,) 200, 100, 50, 25, 12.5, 6.25, and (3.125); or 100, 50, 25, and 12.5 µM; or 100, 66.7, 44.4, 29.6, 19.7, and 13.2 µM in PBST (0.05% Tween 20) with a total concentration of 5% DMSO in 96-well plates. The running buffer was PBST (0.05% Tween 20) containing 5% DMSO. The analytes were injected at a flowrate of 100 µL/min, contact time was 60 s, and dissociation time was 60–120 s. Solvent correction was performed continuously during the experiments to account for bulk response of DMSO. The data was then baseline-aligned, processed by channel referencing with the blank channel, and solvent-corrected. The equilibrium dissociation constant (K_D_) values were determined from equilibrium analysis in the ProteOn Manager software^TM^. Solubility problems frequently hampered K_D_ determination since the small molecules could not always be dissolved in concentrations high enough to reach a plateau. The K_D_ values in Table 1 consist of an averaged K_D_ ± standard deviation calculated from at least three independent experiments (except from substances where no mean K_D_ or standard deviation of K_D_ is given) from one of the channels. Standard deviation was calculated using √((∑(x − M)^2^)/(*n* − 1)), where x refers to the individual data points, M is the mean, and *n* is the number of data points.

### 2.3. ^1^H NMR Binding Experiment

**Figure 3:**
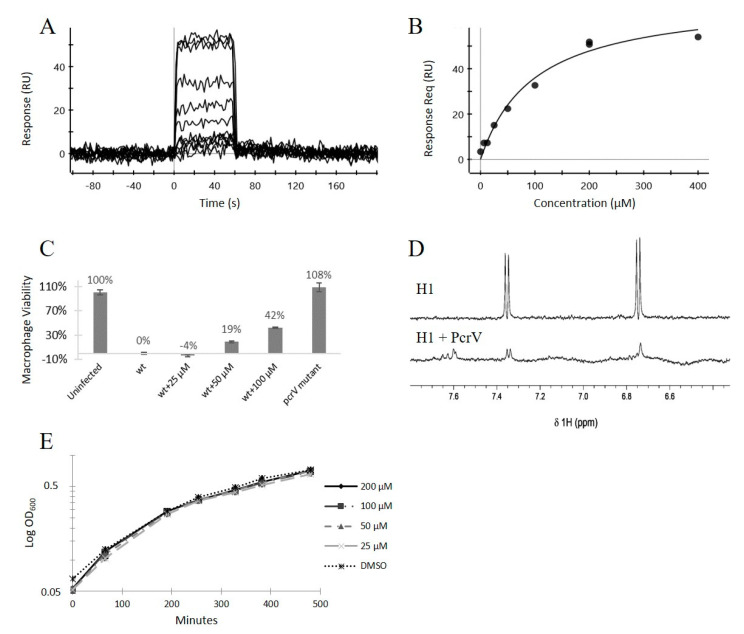
(**A**) Surface plasmon resonance data for compound **H1** binding to PcrV at 400, 200 (duplicate), 100, 50, 25, 12.5, 6.25, and 0 µM. (**B**) Equilibrium analysis of compound **H1** from binding levels at 45-55 s in **A**: K_D_ was determined to be 98.3 µM. R_max_ was 71.3 RU, and Chi^2^ was 10.0 RU. (**C**) Infection assay with dose-response analysis of compound **H1** that protects macrophages from T3SS-mediated *P. aeruginosa* toxicity (wt): uninfected cells and infection with a *pcrV* mutant were used as controls, and standard deviation was calculated by Gaussian approximation. (**D**) ^1^H NMR relaxation-edited binding experiment showing the aromatic region for compound **H1** at 100 µM with (bottom trace) and without 10 µM PcrV (upper trace): equilibrium analysis was performed in the ProteOn Manager software^TM^ (Bio-Rad Laboratories Inc., Hercules, CA, USA). (**E**) Bacterial growth assay where *P. aeruginosa* was grown in the presence of compound **H1** at 200, 100, 50, and 25 µM and OD600 was measured regularly for 8 h: each value represents the mean value of a triplicate; to the control wells, 1% DMSO was added (DMSO). The panels show one representative experiment.

A solution containing 100 µM of the small molecule was prepared from 20 mM stock solution of the tested compound in DMSO and diluted in PBS (pH 7.4) containing trimethylsilyl propanoic acid (TMSP) as an internal standard. This solution was then split into halves and used for preparation of the test and the reference samples as follows: the test sample was prepared by adding 24 µL of 0.211 mM PcrV protein stock solution, resulting in a final protein concentration of 10 µM, whereas the reference sample was prepared by adding the corresponding volume of PBS buffer only before being transferred to 5-mm NMR tubes. The relaxation-edited experiments shown in Figure 3D were recorded on Bruker 600 MHz, and data were acquired at 298 K. Data from 256 scans were accumulated, and a Carr-Purcell-Meiboom-Gill (cpmg) spin-lock of 200 ms was used. Excitation sculpture was applied for water suppression.

### 2.4. Cell Viability Assay

**Figure 4:**
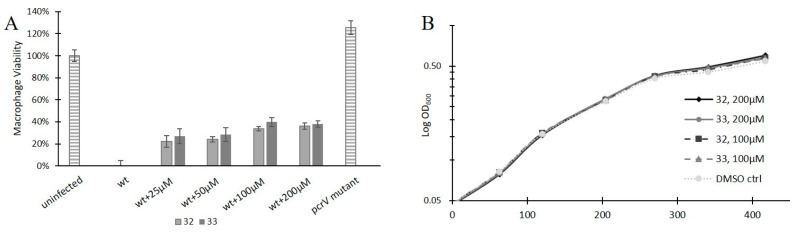
Compounds **32** and **33** inhibit cell infection without affecting bacterial growth. (**A**) Infection assay with dose-response analysis of compound **32** and **33** that protects macrophages from T3SS-mediated *P. aeruginosa* toxicity (wt): uninfected cells and infection with a *pcrV* mutant were used as controls, and standard deviation was calculated by Gaussian approximation. Each value represents the mean value of a triplicate. (**B**) Bacterial growth assay where *P. aeruginosa* was grown in the presence of compounds **32** and **33** at 200, 100, 50, and 25 µM and OD600 was measured regularly for 8 h: each value represents the mean value of a triplicate; to the control wells, 1% DMSO was added (DMSO).

The cell viability assay was performed essentially as described before [35]. J774 cells were seeded in 96-well micro-titer plates (Nunc), 100 µL/well, to a final number of 50,000 cells/well in Dulbecco’s modified Eagle’s medium (DMEM) with glutamax (Merck) supplemented with 10% fetal calf serum (FCS) and 3 µg/mL gentamicin and incubated for 16-18 h. Overnight cultures of the *P. aeruginosa* strains PAK and PAK*pcrV* [10] were grown in Luria Broth (LB) on a rotary shaker at 220 rpm at 37 °C. Prior to infection, the bacteria were diluted 1:10 in DMEM + glutamax and incubated for 1 h at 37 °C (shaking). The J774 cells were washed once with PBS, then 30 µL of DMEM with glutamax and 10% FCS supplemented with the potential binders at concentrations of 50, 100, 200, and 400 µM were added to the wells in triplicates. The infection was initiated by adding 30 µL of the diluted bacterial solutions (where the OD_600_ had been diluted to 0.0008) to each well to initiate infection. This infection results in a final bacterial concentration of OD_600_ = 0.0004 and a multiplicities of infection (MOI) of approximately 8, with final concentrations of the compounds at 25, 50, 100, and 200 µM in 60 µL of DMEM + glutamax and 5% FCS. In the primary screening, the compounds were run at one concentration (100 µM) in duplicates. As an infection control, the wild-type strain PAK with 1% DMSO was added, and as a positive control, PAK*pcrV* with 1% DMSO was added into four wells each. After 3 h and 30 min, 10 µL of UptiBlue Viable Cell Counting Kit (Interchime, France) was added to all wells. The infection was followed under the light microscope and the fluorescence (excitation 535 nm and emission 595 nm) was measured at 4, 5, and 6 h in a microplate reader (Synergy™ H4, Biotek). The results were calculated as percentage of living cells compared to uninfected control, where the uninfected control was set to 100% living cells and the infected control was set to 0% living cells. As a control, compounds were added to uninfected cells in a parallel experiment and viability was calculated as above to screen for cell toxicity. A compound was considered toxic if the viability was <80% of the uninfected control after 6 h of incubation and if a visual change of cell morphology was observed by microscopy. Z’ was calculated for each plate to ensure good quality throughout the screening. Only results from plates with a Z’ > 0.4 were counted. Standard deviation was calculated with Gaussian approximation when appropriate and shown as error bars in Figure 3C and Figure 4A.

### 2.5. Bacterial Toxicity Assay

Overnight culture of the *P. aeruginosa* strain PAK was diluted in LB (Luria Broth) to an OD_600_ of 0.2. The bacteria were added into microtiter plates, and 25, 50, 100, and 200 µM of the potential binders were added in triplicates to the bacteria with a final concentration of 1% DMSO. OD_600_ was measured every hour for seven hours in a microplate reader (Victor, Perkin Elmer). Wells with 1% DMSO without substance were used as positive controls.

## 3. Results and Discussion

### 3.1. Screening and Hit Validation

Amino acid 20-294 of PcrV were cloned and expressed in *Escherichia coli*, and subsequent purification furnished pure PcrV in high yield (Figure S1). The protein was soluble in phosphate-buffered saline (PBS), able to form secondary structures according to circular dichroism (CD) spectroscopy measurements (Figure S2) and to bind to its cognate chaperone, PcrG, using NMR spectroscopy (data not shown), and was therefore considered suitable for SPR screening.

Assay development and screening were performed on a ProteOn XPR36 SPR instrument (Bio-Rad^TM^ Laboratories Inc., Hercules, CA, USA) that offers screening in 96-well plates, allowing analysis of up to 36 different interactions in parallel. The target protein PcrV was immobilized as duplicates on two different channels, and a deactivated control protein, carbonic anhydrase (CAII, Sigma-Aldrich), was immobilized as a negative control on a third channel. This makes it possible to distinguish between selective and nonselective PcrV binders. To allow blank subtraction, a fourth channel was treated in the same way as the other three channels (activation and deactivation) but without protein immobilization. The proteins were immobilized covalently with amine coupling chemistry to carboxylic acid functionalized chips activated with 1-ethyl-3-(3-dimethylaminopropyl) carbodiimide hydrochloride (EDAC) and *N*-hydroxysulfosuccinimide (sulfo-NHS). A library composed of 7600 small organic compounds selected from a ChemBridge diversity set dissolved in dimethylsulfoxide (DMSO) were screened at 100 µM in PBS with 0.05% Tween 20 (PBST) at a final DMSO concentration of 5%. Compounds with two times higher binding to PcrV than to the deactivated control protein CAII were considered as potential binders, while the best binders had more than five times higher binding to PcrV than to CAII, as exemplified in Figure 2. The screening resulted in 395 potential PcrV binders that were tested for biological activity in an infection assay at 100 µM [35].

The T3SS is important for *P. aeruginosa* infection of eukaryotic cells, and a T3SS activator mutant or a mutant lacking the T3SS tip protein PcrV are not able to infect eukaryotic cells [10,15]. The macrophage cell line, J774, was infected with the wild-type *P. aeruginosa* strain PAK, and the potential PcrV binders were added to the wells at a final concentration of 100 µM to screen for inhibition of infection. The T3SS-mediated cytotoxic effect on the eukaryotic cells was measured by using a viability staining method (Uptiblue, Interchim), and the results were measured after 4, 5, and 6 h of infection. The *pcrV*-mutant (PAK*pcrV*) [10], which is not able to infect macrophages due to deletion of the PcrV protein, was used as a control. Out of the 395 tested compounds, 53 inhibited the infection significantly (>3 standard deviations) and were chosen for further validation.

The binding affinity constants (K_D_) for binding of the compounds to PcrV were determined by SPR dose-response experiments as exemplified for **H1** (averaged K_D_ 106 ± 36 µM; Figure 3A,B, Table 1, and Table S1). The 53 compounds were also evaluated in the macrophage cell infection assay at four different concentrations: 200, 100, 50, and 25 µM, as exemplified for **H1** (Figure 3C). The toxicity of the binders was also tested towards *P. aeruginosa* since PcrV binders should function as virulence blockers, i.e., block virulence without affecting bacterial growth (Figure 3E).

To confirm the binding data obtained from the SPR, a secondary qualitative label-free binding experiment based on ^1^H-NMR was performed [50]. In this ^1^H-NMR binding experiment, changes in relaxation time of resonances from small molecules (<600 Da) upon binding to the large protein (PcrV) were observed, as exemplified by the signal reduction for **H1** (Figure 3D).

Taken together, the results from the validation of the binders resulted in 20 compounds that bind the PcrV protein according to NMR spectroscopy and show dose-dependent binding in SPR. In addition, these compounds showed biological activity by inhibiting cell infection with low toxicity towards bacteria and eukaryotic cells. The identity and the purity of the 20 potential PcrV binders that showed efficacy in the cell infection assay were then validated using liquid chromatography mass spectrometry (LCMS). The ChEMBL database [47,51] was used for cheminformatics and bioactivity mining using substructure and similarity searches. Resynthesizing the most promising compounds along with a few analogues and subsequent evaluation in SPR and in the cell infection assay confirmed **H1** (Table 1) as the most promising starting point for further investigation.

### 3.2. Design, Synthesis and Structure–Activity Relationships

**Scheme 1:**
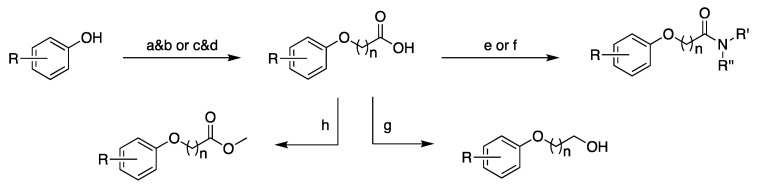
Williamson ether synthesis and modification of the carboxylic acid functionality: the reagents and conditions were (**a**) Br(CH_2_)_n_CO_2_Et, K_2_CO_3_, KI, (CH_3_)_2_CO, reflux, 18–24 h; (**b**) LiOH·H_2_O, tetrahydrofuran (THF)/CH_3_OH/H_2_O (3:1:1), 65 °C microwave irradiation (MWI), 15 min, 54–94% over two steps; (**c**) Br(CH_2_)_n_CO_2_Et, K_2_CO_3_, CH_3_CN, reflux, 24 h; (**d**) NaOH (aq., 1M), THF/CH_3_OH, 40–80% over two steps; (**e**) HATU, R’NHR’’, dimethylformamide (DMF), room temperature (RT), 18–20 h, 47–82%; (**f**) R’NHR’’, 2-(1H-benzotriazole-1-yl)-1,1,3,3-tetramethylaminium tetrafluoroborate (TBTU), DMF, RT, 18–20 h, 40–65%; (**g**) BH_3_.(CH_3_)_2_S, THF, 0 °C-RT, 18 h, 74%; and (**h**) 2,6-Lutidine, Me_3_SiCH_2_N_2_, CH_2_Cl_2_,/CH_3_OH, 0 °C, 1 h, 79%.

**Scheme 2:**
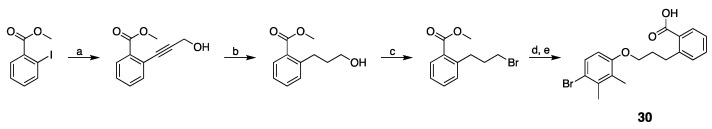
Reagents and conditions: (**a**) Propargyl alcohol, Pd(PPh_3_)_2_Cl_2_ (5 mol%), CuI (3 mol%), triethylamine (TEA)/THF (3:1), 40 °C, microwave irradiation (MWI), 30 min, quant.; (**b**) Pd/C 10%, H_2_, EtOAc (ethyl acetate)/CH_3_OH, RT, 2 h, 59%; (**c**) CBr_4_, PPh_3_, CH_2_Cl_2_, 0 °C, 30 min, 94%; (**d**) 4-bromo-2,3-dimethylphenol, K_2_CO_3_, KI, (CH_3_)_2_CO, reflux, 18 h; and (**e**) LiOH·H_2_O, THF/CH_3_OH/H_2_O (3:1:1), 65 °C, MWI, 15 min, 85% (over two steps).

**Scheme 3:**
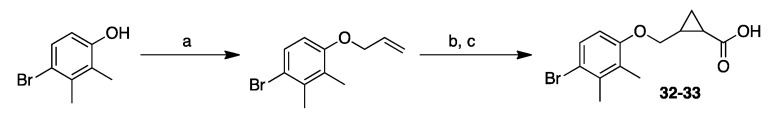
Reagents and conditions: (**a**) Allyl bromide, K_2_CO_3_, KI, (CH_3_)_2_CO, reflux, 18 h, 88%; (**b**) Ethyl diazoacetate, Rh_2_(OAc)_2_ (1 mol%), CH_2_Cl_2_, RT, 18 h, 68% overall yield (2:1 racemic *E:Z* diastereoisomers which were separated by chromatography); and (**c**) LiOH·H_2_O, THF/CH_3_OH/H_2_O (3:1:1), 65 °C, MWI, 15 min, 78% (*Z*) and 27% (*E*) after preparative high-performance liquid chromatography (HPLC).

To probe the binding interactions between **H1** and PcrV and to establish structure-activity relationships, a number of analogues were designed and synthesized as outlined in Scheme 1, Scheme 2 and Scheme 3.

The central ether linker in all compounds **1**–**33** were synthesized via Williamson ether synthesis from the corresponding phenol derivatives in 40–94% yields as outlined in Scheme 1. Compounds **18**–**27** with terminal amide or sulfonamide were synthesized from the corresponding acid and amine using 1-[bis(dimethylamino)methylene]-1*H*-1,2,3-triazolo[4,5-*b*]pyridinium 3-oxid hexafluoro- phosphate (HATU) or 1*H*-benzotriazole-1-yl)-1,1,3,3-tetramethylaminium tetrafluoroborate (TBTU) as coupling agents (Scheme 1). Alcohol **28** and ester **29** were prepared from the corresponding acid via reduction with borane or esterification with trimethylsilyldiazomethane, respectively. Compound **30** was synthesized by employing Sonogashira coupling and alkyne reduction followed by Appel reaction and *O*-alkylation (Scheme 2).

Compounds **32** and **33** with cyclopropyl linker were synthesized from the corresponding allyloxy substrate by applying intermolecular carbenoid cyclopropanation reaction with dirhodium tetraacetate and ethyl diazoacetate [52] that afforded the desired substance in a diastereomeric mixture in 2:1 (*E/Z*) ratio, which was subjected to chromatography that allowed the separation of the *E* and *Z* diastereoisomers in their racemic form and an overall yield of 68% (Scheme 3).

All analogues were evaluated for dose-dependent binding to PcrV using SPR (Table 1); compounds with dose-dependent binding were tested for virulence blocking activity in the macrophage infection assay and for toxicity towards bacteria and eukaryotic cells.

***Substitution***: Based on the possibility that the aromatic ring of **H1** is involved in a hydrophobic or halogen interaction [53], we designed and synthesized analogues with various substituents on different positions of the aromatic ring. We observed that dihalogenated aromatics with a hydrophobic character such as 2,4-dichloro (**1**) or 3,5-dicholoro (**2**) showed binding affinities < 127 µM, while the 4-chloro (**3**) and 4-iodo (**4**) or 4-bromo-2-fluoro (**5**) showed no binding at the examined concentrations. Electron-donating groups with a resonance effect located *ortho* (**6**), *meta* (**7**), or *para* (**8**) to the linker impaired binding. Analogues carrying one electron withdrawing group in the *meta* (**9**) position as well as polar functionalities with the capacity to form hydrogen bonds such as carboxylic acid (**10**) showed no binding to PcrV. The same result was obtained with a heteroaromatic compound (**11**). Taken together, these results suggest a preference for an aromatic ring with hydrophobic and electron withdrawing substituents. Accordingly, a few trisubstituted analogues were synthesized by shuffling the dimethyl groups around the ring (**12**, **13**, and **14**) while keeping a bromo or cyano substituent in the *para* position. The 2,6-dimethyl pattern (**12** and **13**) reduced binding, while hydrophobic substituents on positions 2,3 (**H1**) or 3,5 (**14**) resulted in binding affinities < 150 µM. To explore the possibility of extending the aromatic group further, we tested a larger substituent, such as *p*-cyclohexyl (**15**), benzyl (**16**), or phenoxy (**17**) on the aromatic ring. Only the *p*-cyclohexyl substituent was tolerated with K_D_ of 153 µM (**15**).

***Carboxylic acid functionality:*** To study the importance of the carboxylic acid moiety and the possibility to extend the structure via this functionality, we synthesized analogues with amide (**18**–**24**), sulfonamide (**25**–**27**), alcohol (**28**), ester (**29**), and aromatic carboxylic (**30**) functionalities. Analogues with carboxamide (**18**) or *N*-methyl carboxamide (**19**) showed improved or similar binding affinity to the hit carboxylic acid (**H1**). *N*-substituted amides carrying carboxylic acid at different spatial arrangements such as racemic nipecotic acid (**20**), phenylalanine (**21**), pipecolic acid (**22**), isonipecotic acid (**23**), 2-aminobenzoic acid (**24**), and *ortho*-linked benzoic acid (**30**) showed binding with K_D_ values in the range of 62 to 131 µM with the exception of **24** that failed to bind PcrV. The underlying reason might be its ability to form a stable intramolecular hydrogen bond with the amide linker, as noted in the ^1^H-NMR spectroscopy for this compound, thus affecting its overall conformation. Interestingly, the **H1** analogue with an ester functionality (**29**) that lacks the ionic head and the hydrogen bonding was capable of binding to PcrV with K_D_ of 110 µM. A similar result was obtained with the corresponding alcohol (**28**) with K_D_ of 127 and 351 µM, respectively. Acyl sulfonamide isosteres (**25**–**27**) showed affinities with K_D_ in a range of 93–164 µM.

***Linker***: A small set of compounds in which we varied the length and conformation of the linker carrying the carboxylic acid moiety were synthesized. A one-atom longer linker (**31**) showed similar affinity to PcrV (K_D_ 120 and 138 µM, respectively) as the Hit compound **H1**, whereas a conformationally locked linker with a racemic *Z* or *E*-cyclopropyl group (**32** and **33**) showed improved affinity with K_D_ of around 60 µM. Full exploration of the potential of linker optimization would be facilitated by access to the structure of PcrV.

### 3.3. Anti-Virulence Properties

All analogues that showed dose-dependent responses in the SPR binding experiments were then evaluated for their anti-virulence efficacy in the infection assay, and their inhibitory effect were calculated (Table 1). Many analogues displayed no or modest anti-virulence activity without clear dose-response (e.g., **20** and **31**), and for some, this could be linked to eukaryotic cell toxicity (e.g., **18** and **19**) (<80% viability compared to DMSO control) or assay interference due to autofluorescence (e.g., **15** and **22**). However, a set of compounds proved to be as efficacious as or better than **H1** such as the close analogue **14**, the carboxylic acid isostere **25**, and compounds **32** and **33** with a cyclopropyl modified linker. Importantly, the most promising analogues **32** and **33** display SPR K_D_ around 61–63 µM and dose-dependent inhibition of the infection up to 38% at 200 µM (Figure 4). Furthermore, this data suggest that stereochemistry can be explored to further optimize efficacy. Compound **1** is a typical chlorophenoxy herbicide with adverse effects in humans, and it is possible that the toxic effects observed for some of the compounds can be traced to this similarity [54].

Nevertheless, a clear correlation between binding affinity and efficacy in the infection model could not be established. The biophysical SPR method is simplified in comparison to the infection model where PcrV is present in its native form as a T3SS multiprotein complex in a context involving both living bacteria and eukaryotic cells. The infection assay is also affected by the cell toxicity of the substance and serum binding, while the binding assay is not; this might also affect the correlation between the results for some of the compounds.

## 4. Conclusions

In this study, we develop and describe a screening funnel to identify nontoxic PcrV binders that block T3SS-mediated virulence in a *P. aeruginosa* cell infection assay as well as to perform a structure-activity relationship analysis with the aim to increase the potency and to lower the toxicity of the hit compound. The analysis indicates the importance of an appropriately substituted aromatic ring of the hit compound **H1** and that the linker and carboxylic acid functionality can be explored to reach more potent nontoxic compounds with anti-virulence properties. Importantly, the compounds are small in size, display good aqueous solubility, are amendable to medicinal chemistry, and could be considered as good starting points for further development. Continued optimization would greatly benefit from access to the PcrV structure. These compounds thus hold promise to be further developed into compounds with efficacy in vivo. Ultimately, small molecule virulence blocking agents targeting PcrV can be used in preventive care, as standalone therapy or in combination with conventional antibiotics, and can thereby improve our capabilities to treat *P. aeruginosa* infections.

## Figures and Tables

**Table 1:** K_D_ and biological evaluation of the synthesized compounds.

ID	Structure	SPR K_D_ (µM) ^a^	Viability Assay (% of Uninfected Control)			
			200 µM	100 µM	50 µM	25 µM
**H1**	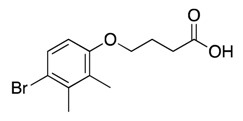	106 ± 36	28%	27%	20%	12%
**1**	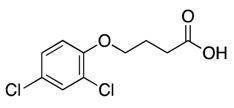	95 ± 41	16% ^b^	21%	12%	14%
**2**	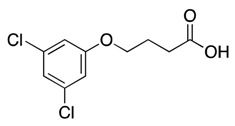	102, 127	nd			
**3**	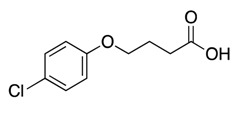	>600	nd			
**4**	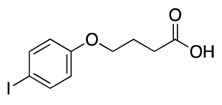	>600	nd			
**5**	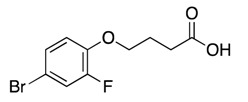	>600	nd			
**6**	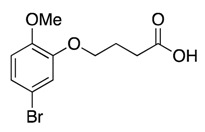	>600	nd			
**7**	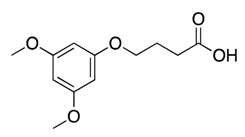	>600	nd			
**8**	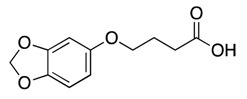	>600	nd			
**9**	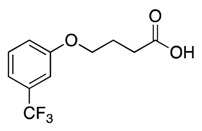	>600	nd			
**10**	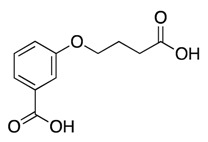	>600	nd			
**11**	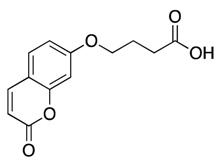	>600	nd			
**12**	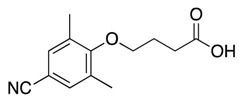	>600	nd			
**13**	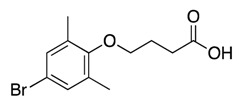	431 ± 110	20%	21%	18%	19%
**14**	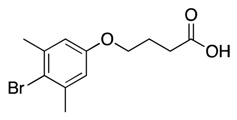	148 ± 62	32%	26%	23%	13%
**15**	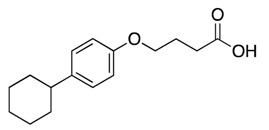	153	17% ^c^	24% ^c^	17% ^c^	12% ^c^
**16**	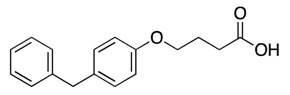	>600	nd			
**17**	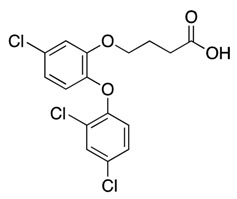	536	−2% ^b^	7%	3%	10%
**18**	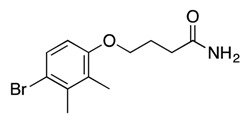	42 ± 12	Toxic ^e^			
**19**	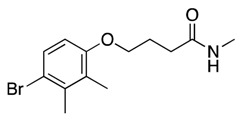	121 ± 45	Toxic ^e^			
**20**	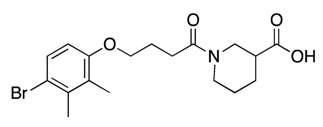	71, 131	9%	15%	15%	15%
**21**	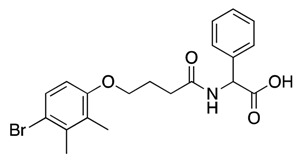	67, 88	30% ^c^	36% ^c^	26% ^c^	12% ^c^
**22**	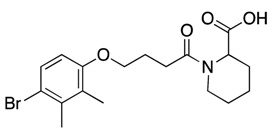	62, 77	28% ^c^	29% ^c^	27% ^c^	18% ^c^
**23**	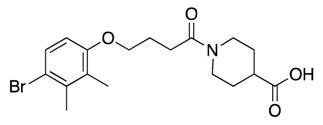	94, 103	9% ^b^	20%	18%	17%
**24**	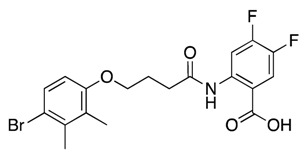	>600	nd			
**25**	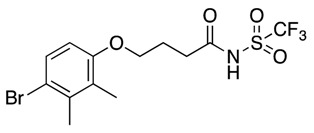	102, 164	34%	22%	11%	9%
**26**	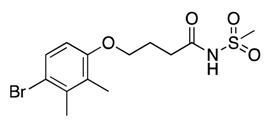	93 ± 31	13%	17%	11%	10%
**27**	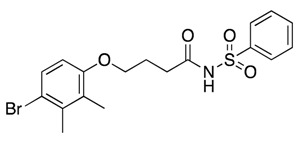	118 ± 62	−6% ^b^	25%	15%	13%
**28**	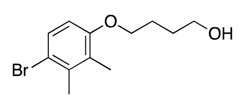	127, 351	Toxic ^e^			
**29**	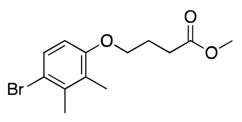	110 ± 83	14% ^c^	23% ^c^	12% ^c^	8% ^c^
**30**	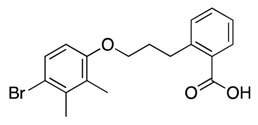	113 ± 31	−16% ^b^	21%	25%	16%
**31**	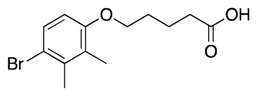	120, 138	17%	18%	17%	11%
**32** ^d^	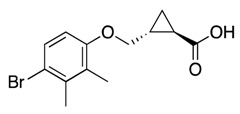	61 ± 10	38%	33%	21%	22%
**33** ^d^	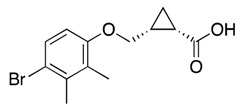	63 ± 4	36%	38%	24%	24%

nd: not determined. ^a^ K_D_ has been determined by equilibrium analysis in the ProteOn Manager software^TM^ (Bio-Rad Laboratories Inc.). K_D_ values consists of a mean K_D_ ± standard deviation (except from substances with duplicates runs or less) calculated from three or more independent experiments. Standard deviation was calculated using √((∑(x−M)^2^)/(*n*−1)), where x refers to the individual data points, M is the mean, and *n* is the number of data points. For compound where *n* < 3, the individual K_D_ values are given. ^b^ The compound is toxic^e^ towards eukaryotic cells, which might affect the results negatively. ^c^ The compound is fluorescent, which might affect the results positively. ^d^ Racemic compound. ^e^ <80% viability compared to the DMSO control.

## Data Availability

Data is contained within the article and the Supplementary Materials.

## Supplementary Materials

The following are available online at https://www.mdpi.com/2218-273X/11/1/55/s1, Figure S1: Purification of PcrV, Figure S2: Stability of PcrV, Table S1: SPR dose-response curves for compounds with determined KD, Table S2: cLogP for all compounds, buffers, and medium used during protein purification chemical synthesis, general chemistry, synthetic methods, and analytical data for all compounds.
