# Supplementary material for: Identification of Small Molecules Blocking the Pseudomonas aeruginosa Type III Secretion System Protein PcrV
Source: Biomolecules. 2021 Jan 4;11(1):55. doi: 10.3390/biom11010055 (PMC7824769; doi:10.3390/biom11010055)
Supplement: Supplementary file 1 [file biomolecules-11-00055-s001.pdf]

Supporting information

Identification of small molecules blocking the *Pseudomonas aeruginosa* type III secretion system protein PcrV

Charlotta Sundin[1,*], Michael Saleeb[1], Sara Spjut[1], Liena Qin[1], Mikael Elofsson[1]

[1]Department of Chemistry, Umeå University, Umeå, Sweden

e-mail address corresponding author: charlotta.sundin@umu.se

Contents:

**S2**	**:**	**Figure S1**	Purification of PcrV.
**S3**	**:**	**Figure S2**	Stability of PcrV.
**S4**	**:**	**Figure S3**	Bacterial growth curves
**S5-S10**	**:**	**Table S1**	SPR dose-response curves for compounds with determined K_D.
**S11**	**:**	**Table S2**	cLogP for all compounds.
**S12-S13**	**:**	Buffers and medium used during protein purification.	
**S14-S23**	**:**	General chemistry, synthetic methods and analytical data for all compounds.	

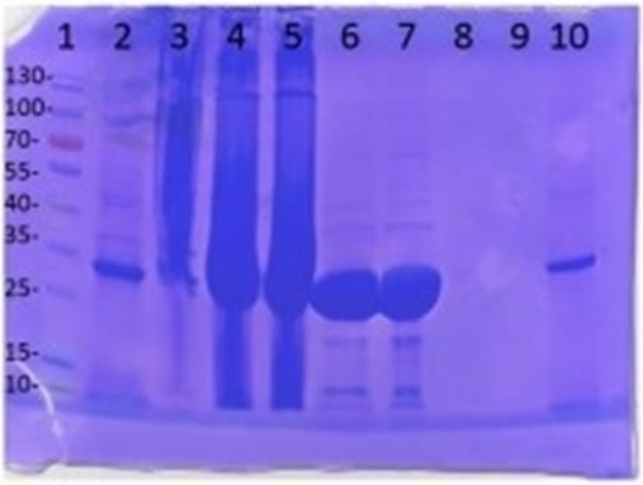

1. kDa Ladder, prestained
2. Total sample after sonication
3. Pellet after sonication
4. Supernatant after sonication
5. Flow through
6. Eluate
7. Eluate after dialysis
8. Empty
9. Empty
10. Total sample before sonication

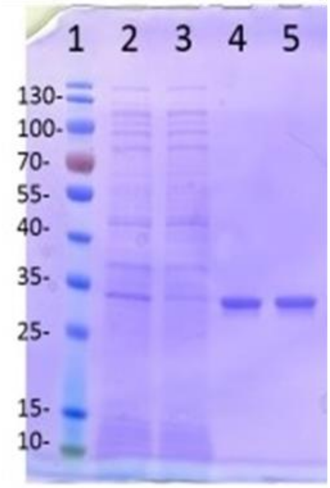

1. kDa Ladder, prestained
2. Total sample after sonication, batch 1
3. Total sample after sonication, batch 2
4. Eluate after dialysis diluted 50x, batch 1
5. Eluate after dialysis diluted 50x, batch 2

Figure S1. Purification of PcrV. The samples were mixed with sample buffer and loaded (10 µl) on to a 12 % sodium dodecyl sulfate (SDS) polyacrylamide gel and the proteins were visualized using Coomassie staining. PcrV could be seen just below 35 kDa in size. Upper gel shows undiluted batch 1 and lower gel shows diluted samples of batch 1 and 2. Batch 1 was used for the SPR-screening campaign.

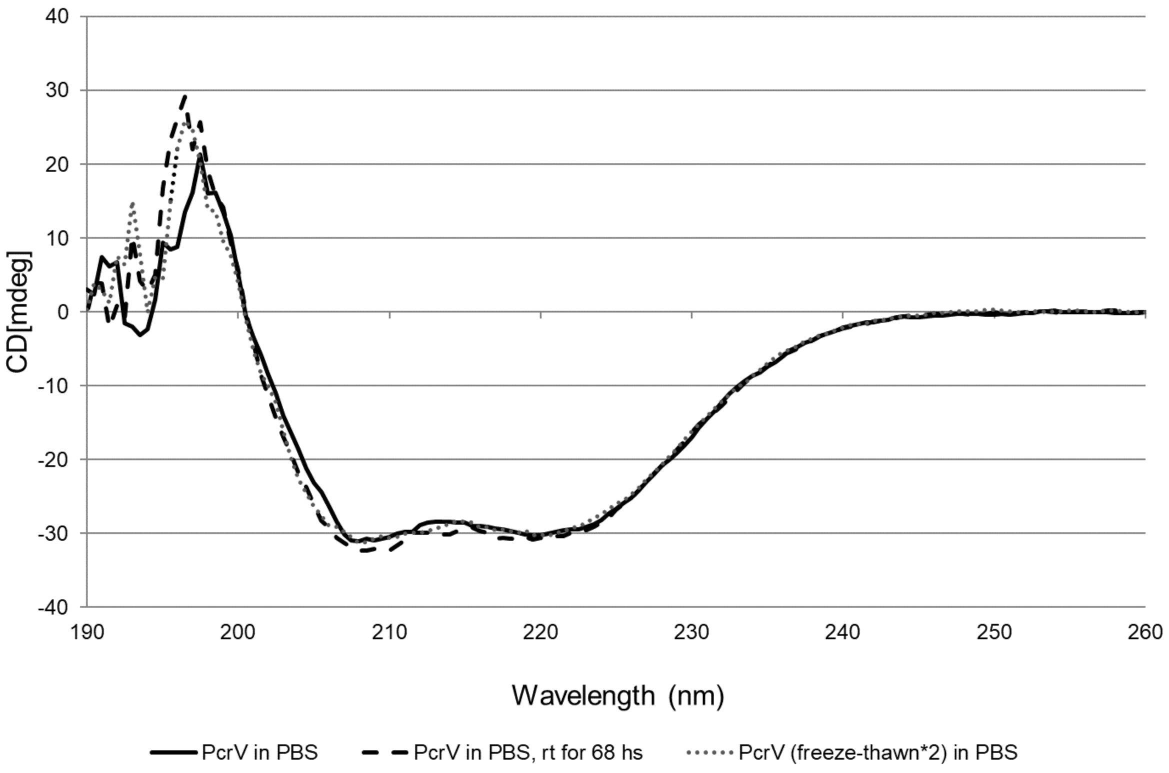

Figure S2. Stability of PcrV (10 µM in PBS pH 7.4) was studied using CD-spectroscopy (J715 Spectropolarimeter, JASCO).

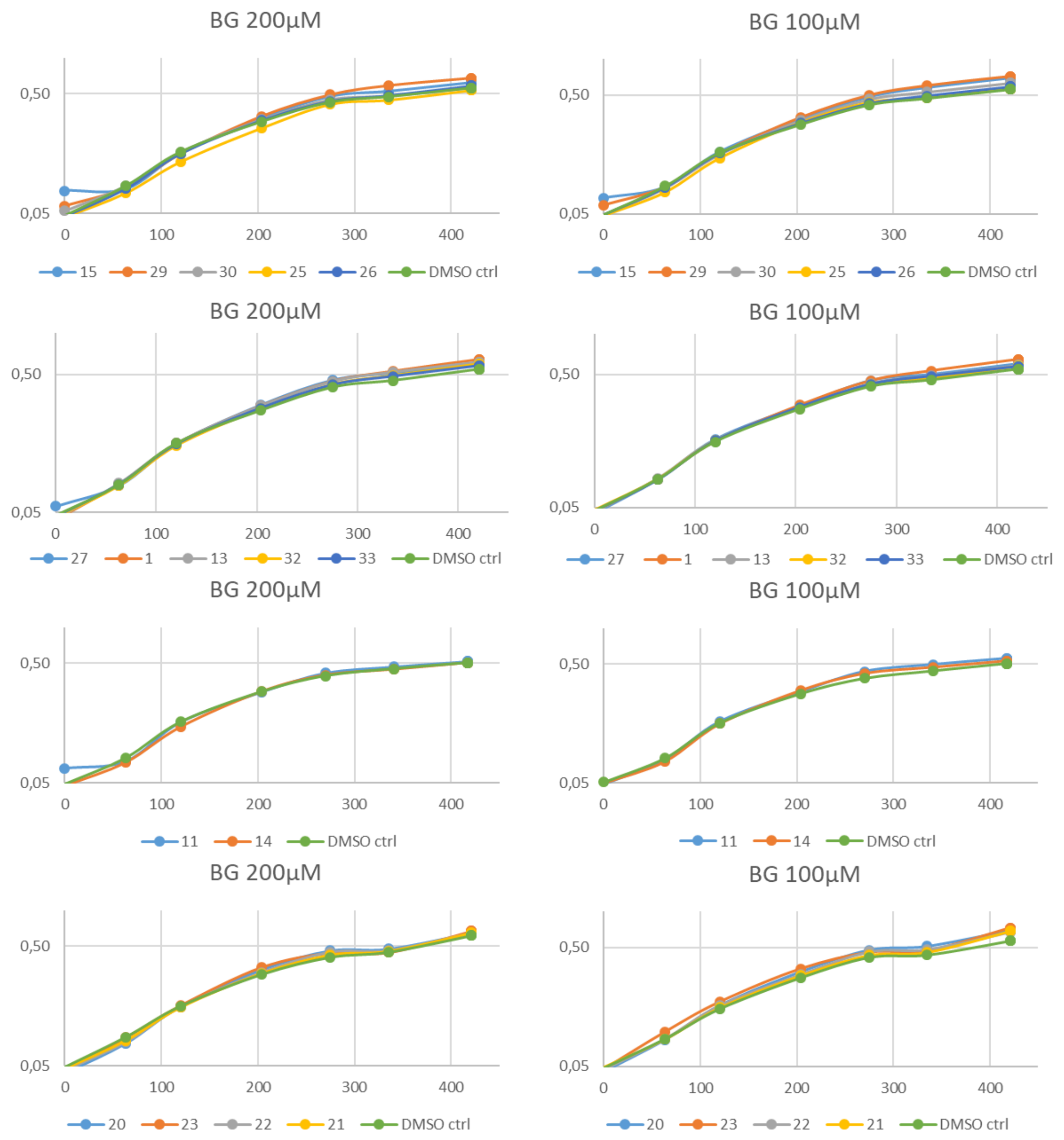

Figure S3. Bacterial growth assay where *P. aeruginosa* was grown in the presence of compounds at 200 and 100 µM and OD_{600} was measured regularly for 7 h. Each value represents the mean value of a triplicate, to the control wells 1 % DMSO was added (DMSO ctrl).

Table S1. SPR dose-response curves for compounds with determined K_D, typically < 150 µM from a single experiment. * Standard error of K_D is calculated by the ProteOn Manager software (Bio-Rad Laboratories Inc.).

ID (Archive no.)	KD±standard error of KD*/ R_{max}/ Chi^2	Concentration interval (uM)	Time interval for equilibrium analysis	Sensorgram	Equilibrium analysis
**1 (ME1044)**	124±53/42.2/6.1	200/150/150/113/84.4/63.3/47.5/35.6/26.7/20/0	45-55 s	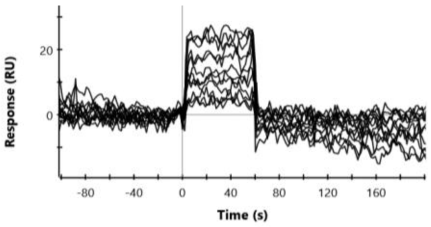	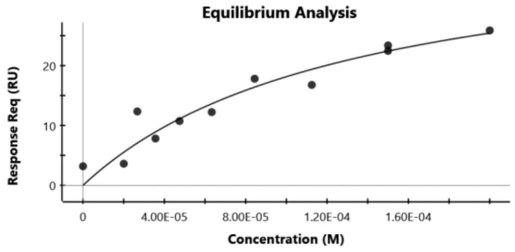
**2 (ME1042)**	127±57/37.4/6.6	200/150/150/113/84.4/63.3/47.5/35.6/26.7/20/0	45-55 s	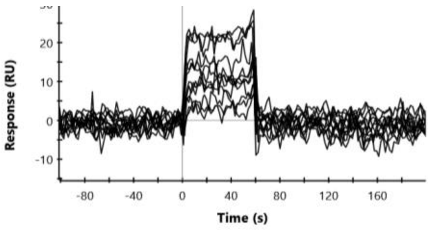	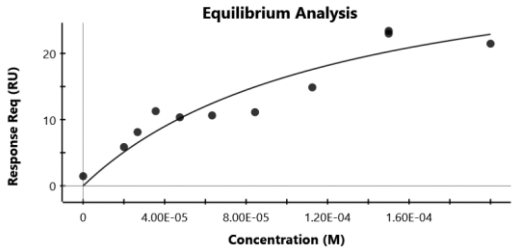
**14 (ME0941)**	80.7±30.3/78.9/38	200/150/150/113/84.4/63.3/47.5/35.6/26.7/20/0	45-55 s	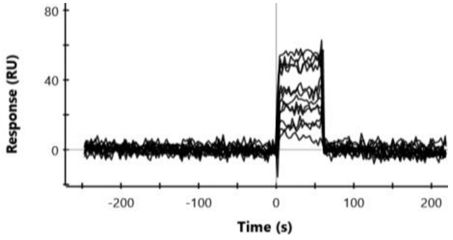	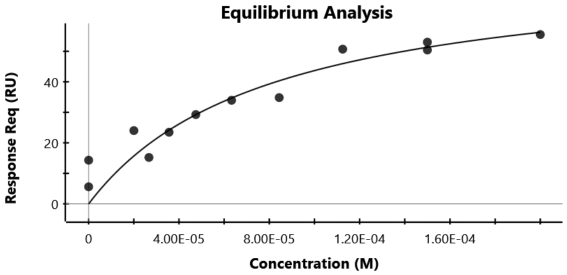

**Table S1.** SPR dose-response curves for compounds with determined K_D, typically < 150 µM from a single experiment. * Standard error of K_D is calculated by the ProteOn Manager software (Bio-Rad Laboratories Inc.).					
**ID (Archive no.)**	**KD±standard error of KD*/ R_{max}/ Chi^2**	**Concentration interval (uM)**	**Time interval for equilibrium analysis**	**Sensorgram**	**Equilibrium analysis**
**15 (ME0988)**	153±4.9/81.7/6.6	200/100/100/50/25/0	5-15 s	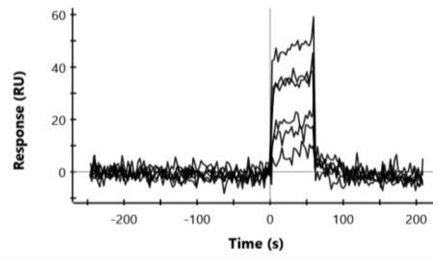	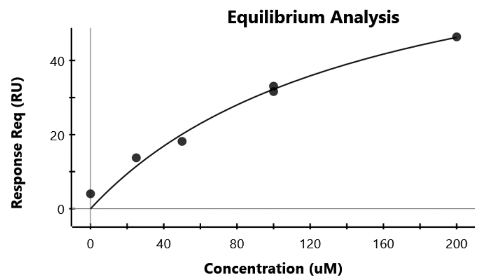
**18 (ME1005)**	32.9±5/50.7/5.7	200/150/150/113/84.4/63.3/35.6/26.720/15/0	45-55 s	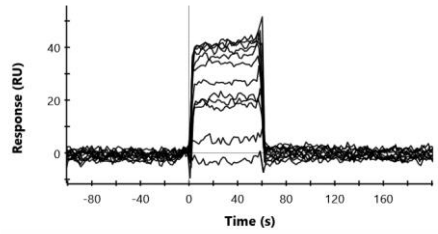	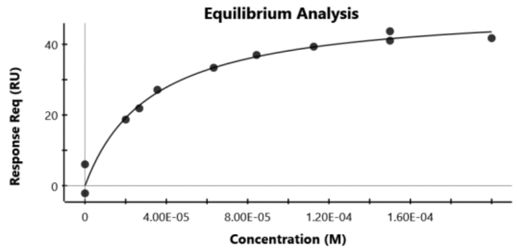
**19 (ME0987)**	177±31.6/122.8/6.3	200/150/150/113/84.4/63.3/47.5/35.6/26.7/20/0	5-15 s	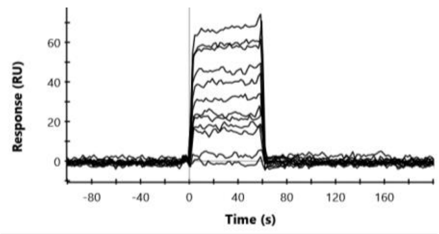	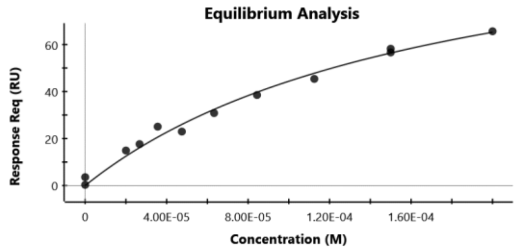

Table S1. SPR dose-response curves for compounds with determined K_D, typically < 150 µM from a single experiment. * Standard error of K_D is calculated by the ProteOn Manager software (Bio-Rad Laboratories Inc.).					
**ID (Archive no.)**	**KD±standard error of KD*/ R_{max}/ Chi^2**	**Concentration interval (uM)**	**Time interval for equilibrium analysis**	**Sensorgram**	**Equilibrium analysis**
**20 (ME1049)**	71.2±23/94.3/10.2	100/50/50/25/12.5	45-55 s	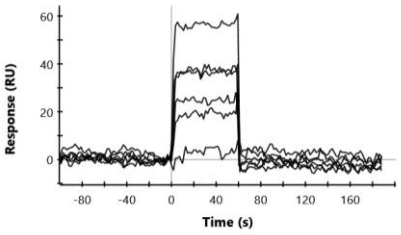	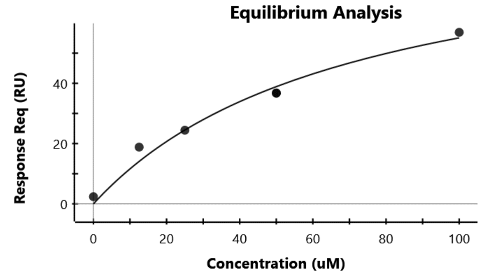
**21 (ME1052)**	66.5±18.5/149.8/20.7	100/50/50/25/12.5/0	5-15 s	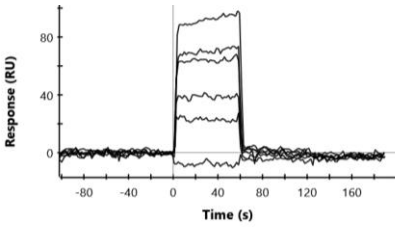	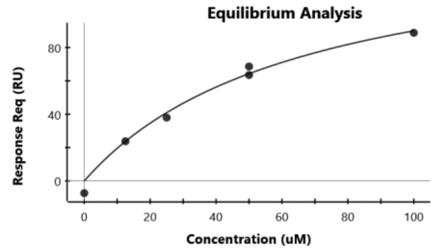
**22 (ME1051)**	61.5±14.9/108.6/9.2	100/50/51/25/12.5/0	5-15 s	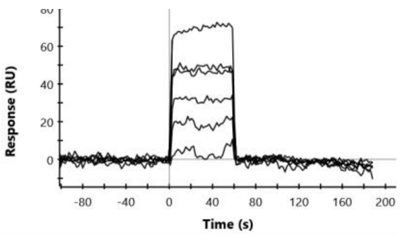	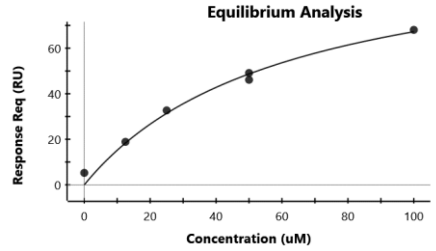

**Table S1.** SPR dose-response curves for compounds with determined K_D, typically < 150 µM from a single experiment. * Standard error of K_D is calculated by the ProteOn Manager software (Bio-Rad Laboratories Inc.).					
**ID (Archive no.)**	**KD±standard error of KD*/ R_{max}/ Chi^2**	**Concentration interval (uM)**	**Time interval for equilibrium analysis**	**Sensorgram**	**Equilibrium analysis**
**23 (ME1050)**	94.1±31/98.1/7.1	100/50/50/25/12.5/0	5-15 s	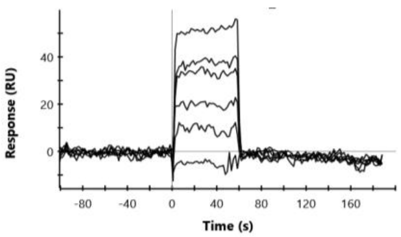	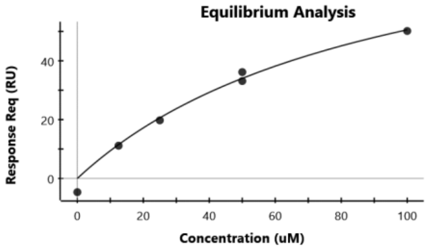
**25 (ME1016)**	102±20/100.8/2.3	100/50/5025/12.5/0	5-15 s	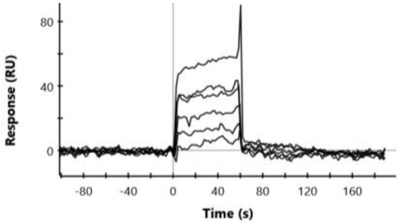	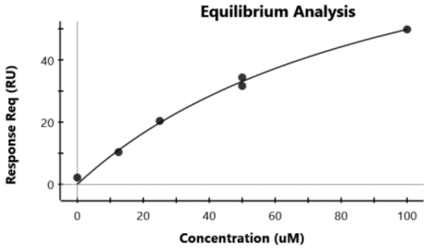
**26 (ME1017)**	135±30/100.7/5.5	150/100/100/66.7/66.7/44.4/29.6/19.7/13.2/0	45-55 s	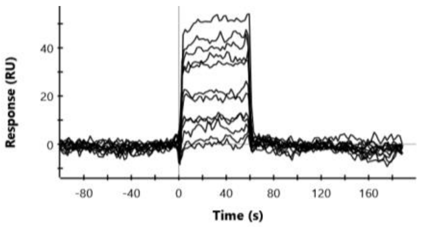	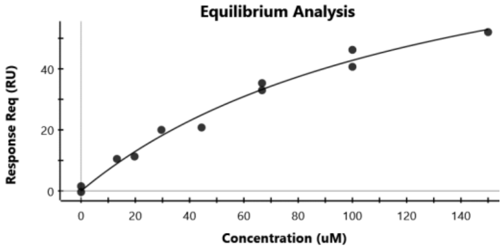
**27 (ME1018)**	116±18/262/47	200/150/150/113/84.4/63.3/47.5/35.6/26.7/20/0	45-55 s	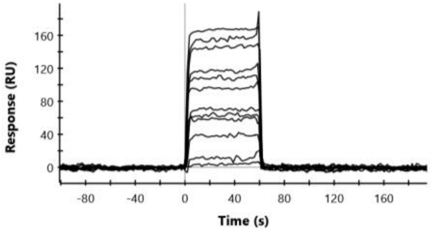	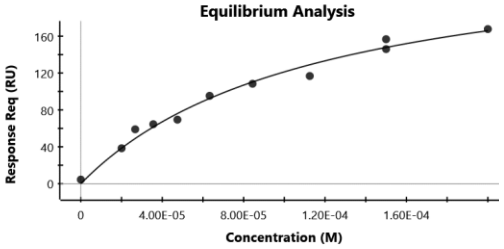

Table S1. SPR dose-response curves for compounds with determined K_D, typically < 150 µM from a single experiment. * Standard error of K_D is calculated by the ProteOn Manager software (Bio-Rad Laboratories Inc.).

ID (Archive no.)	KD±standard error of KD*/ R_{max}/ Chi[2]	Concentration interval (uM)	Time interval for equilibrium analysis	Sensorgram	Equilibrium analysis
**29 (ME0990)**	206±77/60/4.8	200/150/150/113/84.4/63.3/47.5/35.6/26.7/20/0	45-55 s	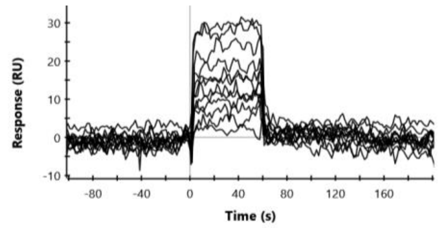	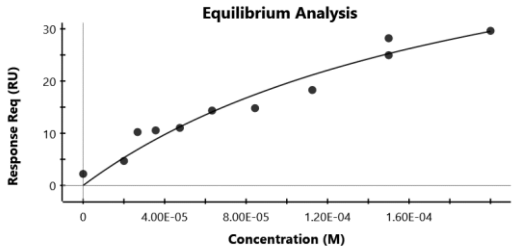
**30 (ME0993)**	149±27/292/50	200/150/150/113/84.4/63.3/47.5/35.6/26.7/20/0	45-55 s	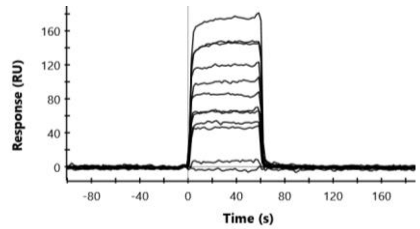	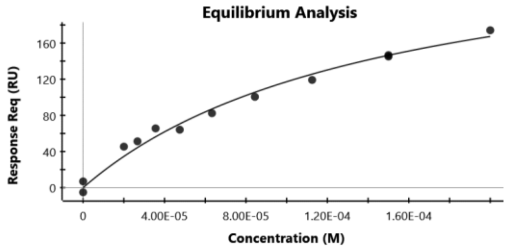
**31 (ME0985)**	138±39/93.7/14.8	200/150/150/113/84.4/63.3/47.5/35.6/26.7/20/0	45-55 s	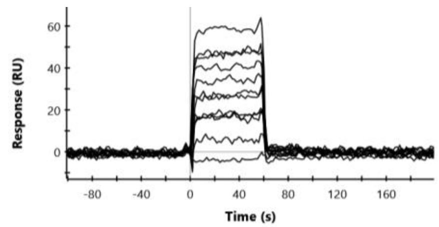	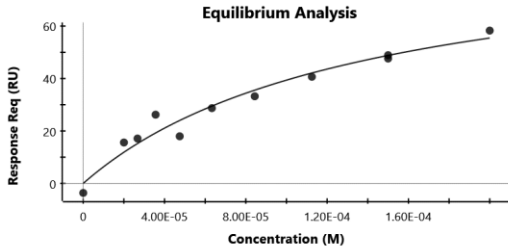

**Table S1.** SPR dose-response curves for compounds with determined K_D, typically < 150 µM from a single experiment. * Standard error of K_D is calculated by the ProteOn Manager software (Bio-Rad Laboratories Inc.).					
**ID (Archive no.)**	**KD±standard error of KD*/ R_{max}/ Chi^2**	**Concentration interval (uM)**	**Time interval for equilibrium analysis**	**Sensorgram**	**Equilibrium analysis**
**32 (ME1047)**	61.1±16.9/ 81.8/7.0	150/100/100/66.7/ 44.4/0	5-15 s	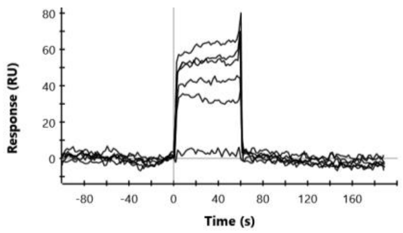	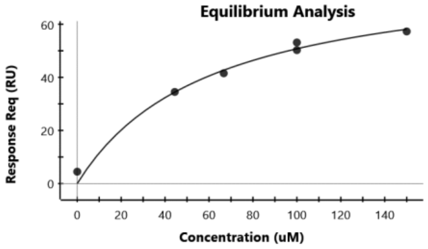
**33 (ME1048)**	59.5±11.1/ 76.8/2.9	100/50/50/25/12.5 /0	45-55 s	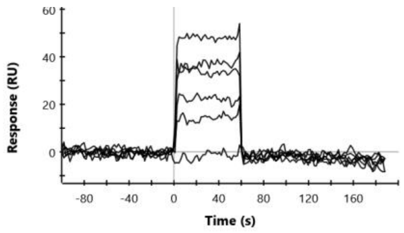	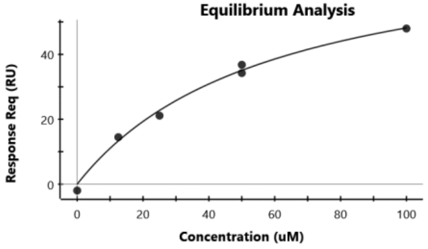

Table S2. cLogP.

ID	Structure	cLogP	ID	Structure	cLogP
**H1**	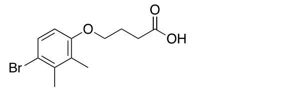	**3.04**	**15**	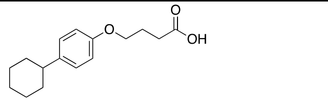	**3.76**
**1**	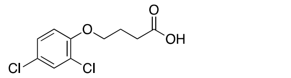	**2.84**	**16**	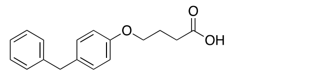	**3.44**
**2**	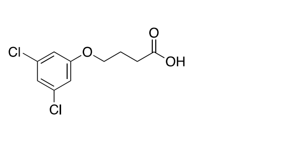	**2.84**	**17**	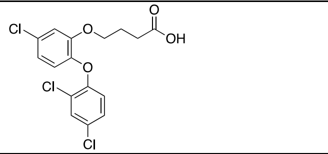	**4.84**
**3**	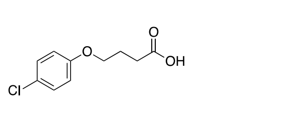	**2.23**	**18**	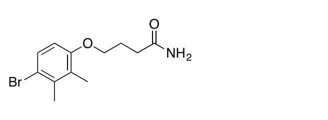	**2.64**
**4**	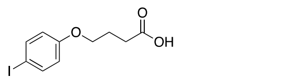	**2.06**	**19**	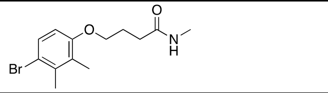	**2.99**
**5**	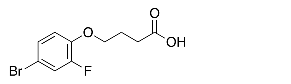	**2.45**	**20**	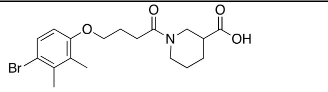	**3.44**
**6**	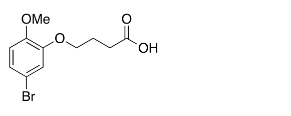	**2.28**	**21**	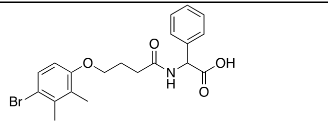	**3.42**
**7**	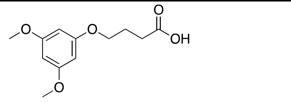	**1.48**	**22**	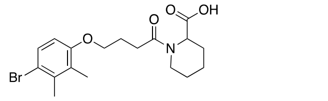	**3.44**
**8**	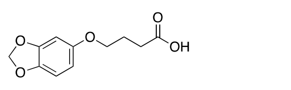	**1.73**	**23**	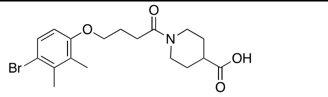	**3.39**
**9**	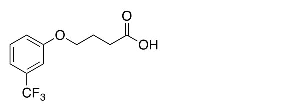	**2.47**	**24**	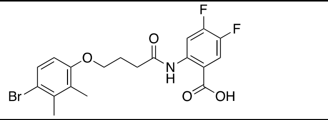	**4.39**
**10**	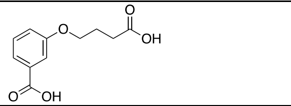	**1.11**	**25**	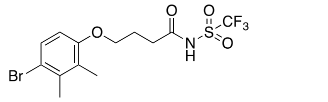	**4.99**
**11**	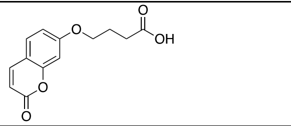	**1.46**	**26**	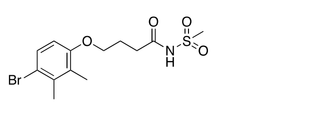	**2.93**
**12**	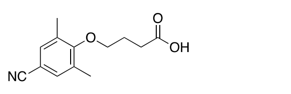	**2.15**	**27**	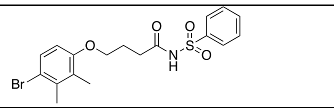	**4.18**
**13**	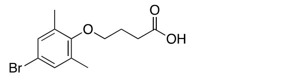	**3.04**	**28**	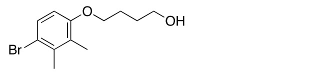	**3.39**
**14**	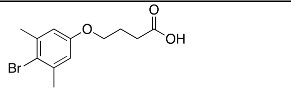	**3.04**	**29**	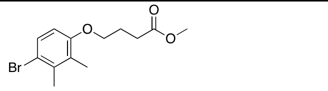	**3.46**
**30**	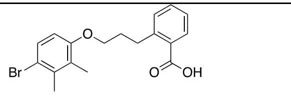	**4.79**	**32**	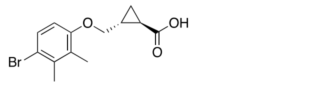	**2.72**
**31**	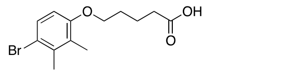	**3.49**	**33**	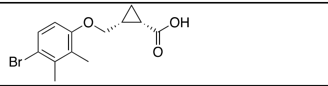	**2.72**

Buffers and medium used during protein purification:

TYM-5052 (1000 ml):

**Total amount**	**1000 ml**
LB	958 ml
$MgSO_4$ (1 M)	1 ml
1000x trace elements mix	1 ml
50x 5052	20 ml
50x M	20 ml
Kanamycin (100 mg/ml)	(50 µg/ml) 500 µl

50x M (100 ml):

13.3 g NH_4Cl (2.5 M)
17 g KH_2PO_4 (1.25 M)
22.25 g Na_2HPO_4 x $2H_2O$ (1.25 M) (17.7 g if Na_2HPO_4)
3.55 g Na_2SO_4 (0.25 M)
Double distillated water up to 100 ml

50x 5052 (100 ml):

25 g glycerol
2.5 g glucose
10 g lactose
Double distillated water up to 100 ml

**Lysis/Wash I:**	Final conc.
Tris pH 8.0 (1M)	20 mM
Imidazole pH 8.0 (1M)	10 mM
NaCl (5M)	150 mM
NP-40	0.2 %
cOmplete, EDTA-free	1 tab to 50 ml
Lysozyme	1 mg/ml
DNAse I	5 µg/ml

Lysozyme and DNAseI for Lysis buffer

**Wash II:**	Final conc.	50 ml	10 ml
Tris pH 8.0 (1M)	20 mM	1 ml	200 µl
Imidazole pH 8.0 (1M)	10 mM	500 µl	100 µl
NaCl (5M)	150 mM	1.5 ml	300 µl

**Elusion buffer:**	Final conc.	50 ml	10 ml
Tris pH 8.0 (1M)	20 mM	1 ml	200 µl
Imidazole pH 8.0 (1M)	330 mM	16.5 ml	3.3 ml
NaCl (5M)	150 mM	1.5 ml	300 µl

1000 x Trace elements: 100 ml stock solution:

Salt	Concentration	g/mg/ml	MW	Special preparation
$FeCl_3.6H_2O$	50 mM	50 ml of 0.1 M stock	270	0.1 M stock (V = 100 ml) 2.7 g in 1:100 diluted HCl
$CaCl_2.2H_2O$	20 mM	0.294 g	147	
$MnCl_2.4H_2O$	10 mM	0.198 g	198	
$ZnSO_4.7H_2O$	10 mM	0.288 g	288	
$CoCl_2.6H_2O$	2 mM	47.6 mg	238	
$CuCl_2.6H_2O$	2 mM	34 mg	171	
$NiCl2.6H_2O$	2 mM	47.5 mg	238	
$Na_2MoO_4.2H_2O$	2 mM	48.4 mg	242	
H_3BO_3	2 mM	400 µl of 0.5 M stock	61.2	0.5 M stock (V= 50 ml) = 1.53 g in ca. 60 mM HCl

General chemistry

Chemicals and reagents were purchased from Aldrich, Alfa Aesar, AK Scientific, Matrix Scientific or Apollo Scientific. Organic solvents were dried using the dry solvent system (Glass Contour Solvent Systems, SG Water USA) except toluene and EtOH, which were dried over activated molecular sieves 3Å. Microwave reactions were performed in Biotage® Initiator$^+$. Flash chromatography was performed on Biotage® Isolera One using appropriate SNAP Cartridge KP-Sil or SNAP Ultra HP-Sphere 25 µm, and UV absorbance at 254 nm. Thin layer chromatography (TLC) was performed on Silica gel 60 F_{254} (Merck) with detection by UV light and/or bromocresol green or Seebach's staining solution unless otherwise is mentioned. Preparative high performance liquid chromatography (HPLC) separation were performed on Gilson System HPLC, using a YMC-Actus Triart C18, 12 nm, S-5 µm, 250 x 20.0 mm, with a flow rate 18 ml/min, detection at 214, 230 or 254 nm and eluent system: (A: 0.75% HCOOH in H_2O, and B: 0.75% HCOOH in CH_3CN) unless otherwise is mentioned. The NMR spectra were recorded at 298 K on Bruker-DRX 400 MHz and 600 MHz and reported in ppm using the residual peak of the solvent DMSO-d_6 (δ_H 2.50 ppm) or $CDCl_3$ (δ_H 7.26 ppm) as internal standard for ^{1}H, and DMSO-d_6 (δ_c 39.50 ppm) and $CDCl_3$ (δ_c 77.16 ppm) as internal standard for ^{13}C. Liquid chromatograpy mass spectrometry (LCMS) was recorded by detecting positive/negative ion (ESI) on Agilent 1290 infinity II connected to DAD and 6130 Quadrupole using Agilent Proshell 120 EC-C18 2.7 µm 3 x 50 mm column and H_2O/CH_3CN (0.1% HCOOH) as the eluent system. High-resolution mass spectroscopy (HRMS) was performed on Agilent 1290 binary LC system connected to an Agilent 6230 Accurate-Mass time-of-flight (TOF) LCMS electro-spray ionization (ESI), calibrated with Agilent G1969-85001 ES-TOF reference mix containing ammonium trifluoroacetate, purine, and hexakis(1H,1H,3H-tetrafluoropropoxy)-phosphazine in 9:1 $CH_3CN:H_2O$. All target compounds were > 95% pure according to HPLC UV-trace, ^{1}H and ^{13}C NMR.

Abbreviations: Ethylacetate (EtOAc), *N,N*-Dimethylformamide (DMF), *N,N*-Diisopropylethylamine (DIPEA), 1-[Bis(dimethylamino)-methylene]-1*H*-1,2,3-triazolo[4,5-*b*]pyridinium 3-oxid hexafluorophosphate or *N*-[(Dimethyl-amino)-1*H*-1,2,3-triazolo-[4,5-*b*]pyridin-1-ylmethylene]-*N*-methylmethanaminium hexafluoro-phosphate *N*-oxide (HATU), , 2-(1H-Benzotriazole-1-yl)-1,1,3,3-tetramethylaminium tetrafluoro-borate (TBTU), Triethylamine (TEA), Tetrahydrofurane (THF).

General procedure for Williamson ether synthesis and ester hydrolysis:

Method A - Exemplified by compound **H1**

To a solution of 4-bromo-2,3-dimethylphenol (584 mg, 2.7 mmol) anhydrous K_2CO_3 (2 equiv., 753 mg, 5.44 mmol) and KI (1.2 equiv., 542 mg, 3.2 mmol) in acetone (12 ml, 0.25 M of the phenol) was added ethyl 4-bromobutyrate (1.2–2 equiv. until completion according to TLC) the mixture was refluxed (not exceeding 80 °C) for 18-24 h and the reaction was monitored by TLC (EtOAc/Heptane 1:3) until the starting phenol was completely consumed. The solid material was filtered off, washed with acetone and the filtrate was concentrated under vacuum to afford the crude product, which was dissolved in THF/MeOH/H_2O (3:1:1, 20 ml) in a microwave vial and LiOH.H_2O (5 equiv., 571 mg, 13.6 mmol) was added. The mixture was heated in a microwave reactor for 15–30 min at 65 °C until completion according to TLC (EtOAc/Heptane 1:1, stained with bromocresol green). Volatile solvents were evaporated under vacuum and the remaining aqueous layer was acidified with HCl (6 M) to pH < 3 and the solid precipitate was collected and dried under vacuum to afford the product in 93% yield as white powder.

Method B - Exemplified by compound **9**

3-Hydroxybenzotrifluoride (16.2 mg, 0.10 mmol), ethyl 4-bromobutyrate (23.4 mg, 0.12 mmol) and K_2CO_3 3-5 equiv was mixed in CH_3CN (1 ml) and stirred in 80°C oil bath for 24 h and the reaction was monitored with LCMS. The reaction mixture was filtered to remove salt and CH_3CN

was removed under vacuum. THF (0.4 ml), MeOH (0.4 ml) and aqueous NaOH solution (1 M, 0.4 ml) were added to the reaction tube. The resulting solution was stirred at room temperature for 3 h. The reaction was monitored with LCMS. The reaction mixture was neutralized with 0.4 ml of HCl solution (1 M). After removal of the organic solvent under vacuum, the product was extracted with CH_2Cl_2 (3 ml x 2) and dried over Na_2SO_4. The pure product was obtained as white solid after silica gel chromatography (Biotage 10 g, MeOH/CH_2Cl_2 5%) in 40% yield.

General procedure for amide coupling:

Method C - Exemplified by compound **20**

Acid derivative (50 mg, 0.17 mmol) and TBTU (73 mg, 0.22 mmol, 1.3 equiv.) were dissolved in anhydrous DMF (0.85 ml, 0.2 M of the acid) before DIPEA (180 µl, 6 equiv.) was added, and the mixture was stirred at room temperature for 30 min. To the above mixture was added a solution of the HCl salt of amino acid ester (43 mg, 0.22 mmol, 1.3 equiv.) in anhydrous DMF (1.1 ml), and the reaction mixture was continued to stir at room temperature for 18 h. LCMS and TLC (CH_2Cl_2/MeOH 5%) showed full conversion of the starting material. The mixture was diluted with EtOAc and washed with HCl (1M), H_2O and brine. The organic layer was dried over Na_2SO_4 and concentrated under vacuum. The crude was dissolved in THF/MeOH/H_2O (3:1:1, 2 ml) and $LiOH{\cdot}H_2O$ (36 mg, 0.87 mmol, 5 equiv.) was added and the mixture was stirred in microwave reactor at 65 °C for 15 min upon which LCMS showed full conversion to acid. The mixture was acidified with HCl (6 M) and concentrated under vacuum. The crude was dissolved in MeOH/DMSO (1:1) and purified by preparative HPLC using CH_3CN/H_2O (20→100%, with (A: 0.75% HCOOH in H_2O, B: 0.75% HCOOH in CH_3CN, 10→100% B over 20 min) to afford the product of interest in 56% yield over two steps.

4-(2,4-dichlorophenoxy)butanoic acid - (1)

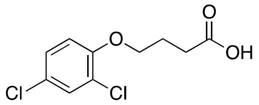

Synthesis: Method A (83% yield, white solid). **^{1}H NMR** (400 MHz, DMSO-d_6): δ_H 12.17 (s, 1H, COO<u>H</u>), 7.54 (d, J = 1.7 Hz, 1H, Ar-<u>H</u>), 7.34 (dd, J = 8.8 Hz, 2.1 Hz, 1H, Ar-<u>H</u>), 7.15 (t, J = 8.8 Hz, 1H, Ar-<u>H</u>), 4.07 (t, J = 5.7 Hz, 2H, C<u>H</u>$_2$), 2.41 (t, J = 7.4 Hz, 2H, C<u>H</u>$_2$), 1.95 (p, J = 6.8 Hz, 2H, C<u>H</u>$_2$). **^{13}C NMR** (100 MHz, DMSO-d_6): δ_C 173.97, 152.91, 129.23, 128.09, 124.38, 122.41, 115.01, 68.04, 29.91, 24.04. **HRMS** (ESI) *m/z*: $[M-1H]^-$ calcd. for $C_{10}H_{10}Cl_2O_3$ 246.9934; observed 246.9940.

4-(3,5-dichlorophenoxy)butanoic acid - (2)

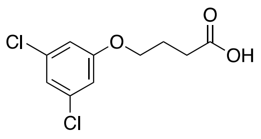

Synthesis: Method A (84% yield, white solid, HPLC purified). **^{1}H NMR** (400 MHz, DMSO-d_6): δ_H 12.17 (s, 1H, COO<u>H</u>), 7.12 (t, J = 1.8 Hz, 1H, Ar-<u>H</u>), 7.03 (d, J = 1.8 Hz, 2H, Ar-<u>H</u>), 4.03 (t, J = 6.5 Hz, 2H, C<u>H</u>$_2$), 2.36 (t, J = 7.3 Hz, 2H, C<u>H</u>$_2$), 1.92 (p, J = 6.9 Hz, 2H, C<u>H</u>$_2$). **^{13}C NMR** (100 MHz, DMSO-d_6): δ_C 174.47, 160.42, 135.05, 120.70, 114.31, 68.09, 30.42, 24.41. **HRMS** (ESI) *m/z*: $[M-1H]^-$ calcd. for $C_{10}H_{10}Cl_2O_3$ 246.9934; observed 246.9945.

4-(4-chlorophenoxy)butanoic acid - (3)

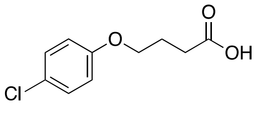

Synthesis: To a solution of sodium (429 mg, 18.6 mmol) in EtOH (18.6 ml, 1 M) was added *p*-chlorophenol (2 g, 15.5 mmol) and the reaction mixture was stirred for 5 min before adding γ- butrylactone (1.43 ml, 18.6 mmol). The reaction mixture was stirred for 5 h at 100 °C after which time the solvent was slowly evaporated by continuous heating to 150 °C for additional 12 h. The remaining brownish solid was dissolved in H_2O, followed by addition of HCl (1 M) until precipitation occurred. The precipitate was isolated by filtration, washed with water and dried under vacuum to yield the title compound in 74% yield as whitish-brownish solid . **^{1}H NMR** (600 MHz, DMSO-d_6): δ_H 12.15 (s, 1H, COO<u>H</u>), 7.31 (d, J = 9.0 Hz, 2H, Ar-<u>H</u>), 6.94 (d, J = 9.0 Hz,

2H, Ar-H), 3.96 (t, *J* = 6.4 Hz, 2H, CH_2), 2.37 (t, *J* = 7.3 Hz, 2H, CH_2), 1.92 (p, *J* = 7.0 Hz, 2H, CH_2). **^{13}C NMR** (150 MHz, DMSO-d_6): δ_C 174.0, 157.3, 129.2, 124.1, 116.1, 66.96, 30.0, 24.1. **HRMS** (ESI) *m/z*: $[M-1H]^-$ calcd. for $C_{10}H_{11}ClO_3$ 213.0324; observed 213.0336

4-(4-iodophenoxy)butanoic acid - (4)

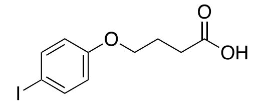

Synthesis: Methods A (87% yield, white solid). **^{1}H NMR** (400 MHz, DMSO-d_6): δ_H 12.14 (s, 1H, COOH), 7.57 (d, *J* = 9.0 Hz, 2H, Ar-H), 6.78 (d, *J* = 9.0 Hz, 2H, Ar-H), 3.95 (t, *J* = 6.4 Hz, 2H, CH_2), 2.36 (t, *J* = 7.3 Hz, 2H, CH_2), 1.91 (p, *J* = 7.0 Hz, 2H, CH_2). **^{13}C NMR** (100 MHz, DMSO-d_6): δ_C 174.5, 158.8, 138.4, 117.7, 83.5, 67.2, 30.5, 24.5. **HRMS** (ESI) *m/z*: $[M-1H]^-$ calcd. for $C_{10}H_{11}IO_3$ 304.9680; observed 304.9684.

4-(4-bromo-2-fluorophenoxy)butanoic acid - (5)

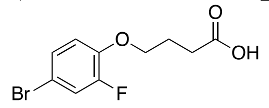

Synthesis: Methods A (54% yield, white solid). **^{1}H NMR** (400 MHz, DMSO-d_6): δ_H 12.17 (s, 1H, COOH), 7.5 (dd, *J* = 10.9 Hz, 2.4 Hz, 1H, Ar-H), 7.31 (dd, *J* = 8.9 Hz, 1.5 Hz, 1H, Ar-H), 7.13 (t, *J* = 9.0 Hz, 1H, Ar-H), 4.05 (t, *J* = 6.5 Hz, 2H, CH_2), 2.37 (t, *J* = 7.3 Hz, 2H, CH_2), 1.94 (p, *J* = 6.9 Hz, 2H, CH_2). **^{13}C NMR** (100 MHz, DMSO-d_6): δ_C 173.8, 116.5, 151.5 (d, $^1J_{CF}$ = 248 Hz), 145.8 (d, $^3J_{CF}$ = 10 Hz), 127.5 (d, $^4J_{CF}$ = 3 Hz), 119.0 (d, $^2J_{CF}$ = 21 Hz), 110.9 (d, $^3J_{CF}$ = 8 Hz), 67.9, 29.8, 23.9. **HRMS** (ESI) *m/z*: $[M-1H]^-$ calcd. for $C_{10}H_{10}BrFO_3$ 274.9725; observed 274.9731.

4-(5-bromo-2-methoxyphenoxy)butanoic acid - (6)

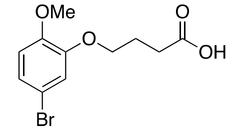

Synthesis: Methods A (83% yield, white solid). **^{1}H NMR** (400 MHz, $CDCl_3$): δ_H 12.17 (s, 1H, COOH), 7.03 (dd, *J* = 8.5 Hz, 2.3 Hz, 1H, Ar-H), 6.99 (d, *J* = 2.3 Hz, 1H, Ar-H), 6.73 (d, *J* = 8.5 Hz, 1H, Ar-H), 4.05 (t, *J* = 6.2 Hz, 2H, CH_2), 3.82 (s, 3H, OCH_3), 2.61 (t, *J* = 7.2 Hz, 2H, CH_2), 2.16 (p, *J* = 6.7 Hz, 2H, CH_2). **^{13}C NMR** (100 MHz, $CDCl_3$): δ_C 178.9, 149.1, 149.0, 124.02, 116.8, 113.2, 112.8, 68.1, 56.2, 30.5, 24.3. **HRMS** (ESI) *m/z*: $[M-1H]^-$ calcd. for $C_{11}H_{13}BrO_4$ 286.9924; observed 286.9929.

4-(3,5-dimethoxyphenoxy)butanoic acid - (7)

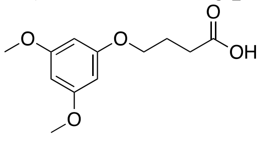

Synthesis: Methods A (77% yield, white solid). **^{1}H NMR** (400 MHz, DMSO-d_6): δ_H 12.14 (bs, 1H, COOH), 6.09 (appear as bs, 3H, Ar-H), 3.94 (t, *J* = 6.5 Hz, 2H, CH_2), 3.7 (s, 6H, 2xOCH_3), 2.37 (t, *J* = 7.3 Hz, 2H, CH_2), 1.91 (p, *J* = 6.9 Hz, 2H, CH_2). **^{13}C NMR** (100 MHz, DMSO-d_6): δ_C 174.2, 161.2, 160.5, 93.3, 93.0, 66.6, 55.2, 30.2, 24.3. **HRMS** (ESI) *m/z*: $[M-1H]^-$ calcd. for $C_{12}H_{16}O_5$ 239.0925; observed 239.0924.

4-(benzo[*d*][1,3]dioxol-5-yloxy)butanoic acid - (8)

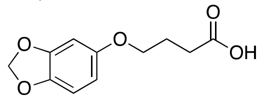

Synthesis: Methods A (76% yield, white solid). **^{1}H NMR** (400 MHz, DMSO-d_6): δ_H 12.13 (s, 1H, COOH), 6.97 (d, *J* = 6.3 Hz, 1H, Ar-H), 6.6 (d, *J* = 2.5 Hz, 1H, Ar-H), 6.34 (dd, *J* = 8.5 Hz, 2.5 Hz, 1H, Ar-H), 5.94 (s, 2H, CH_2), 3.89 (t, *J* = 6.4 Hz, 2H, CH_2), 2.35 (t, *J* =7.3 Hz, 2H, CH_2), 1.88 (p, *J* = 6.9 Hz, 2H, CH_2). **^{13}C NMR** (100 MHz, DMSO-d_6): δ_C 174.0, 153.9, 147.8, 141.0, 108.0, 105.6, 100.9, 97.7, 67.3, 30.1, 24.2. **HRMS** (ESI) *m/z*: $[M-1H]^-$ calcd. for $C_{11}H_{12}O_5$ 223.0612; observed 223.0616.

4-(3-(trifluoromethyl)phenoxy)butanoic acid - (9)

Synthesis: Methods B (40% yield, white solid). **^{1}H NMR** (400 MHz, $CDCl_3$): δ_H 7.38 (t, *J* = 8.1 Hz, 1H, Ar-H), 7.2 (dt, *J* = 7.7 Hz, 0.8 Hz, 1H, Ar-H), 7.11 (t, *J* = 2.1 Hz, 1H, Ar-H), 7.05 (dt, *J* = 8.4 Hz, 2.6 Hz, 1H, Ar-H), 4.06 (t, *J* = 6.1 Hz, 2H, CH_2), 2.61 (t, *J* =7.2 Hz, 2H, CH_2), 2.15 (p, *J* = 6.6 Hz, 2H, CH_2).**^{13}C NMR** (100 MHz, $CDCl_3$): δ_C 178.6, 158.8, 131.8 (d, *J* =31 Hz), 129.9,

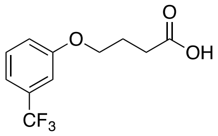

123.9 (q, J = 272 Hz), 117.9, 117.5 (d, J = 3.8 Hz), 111.2 (q, J = 3.6 Hz), 66.7, 30.3, 24.2. **HRMS** (ESI) *m/z*: $[M-1H]^-$ calcd. for $C_{11}H_{11}F_3O_3$ 247.0588; observed 247.0585.

3-(3-carboxypropoxy)benzoic acid - (10)

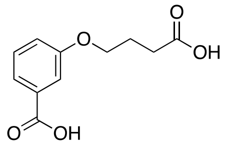

Synthesis: Methods A (86% yield, white solid). **^{1}H NMR** (400 MHz, DMSO-d_6): δ_H 12.56 (bs, 2H, 2xCOOH), 7.57 (dt, J = 7.4 Hz, 0.9 Hz, 1H, Ar-H), 7.47–7.33 (m, 2H, Ar-H), 7.19 (dt, J = 7.8 Hz, 2.5 Hz, 1H, Ar-H), 4.05 (t, J = 6.4 Hz, 2H, CH_2), 2.40 (t, J = 7.3 Hz, 2H, CH_2), 1.96 (p, J = 6.9 Hz, 2H, CH_2). **^{13}C NMR** (100 MHz, DMSO-d_6): δ_C 174.1, 167.1, 158.5, 132.2, 129.7, 121.6, 119.3, 114.5, 66.8, 30.1, 24.2. LCMS (ESI) m/z: $[M-1H]^-$ calcd. for $C_{11}H_{12}O_5$ 223.0612; observed 223.0.

4-((2-oxo-2*H*-chromen-7-yl)oxy)butanoic acid - (11)

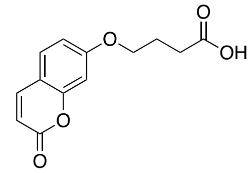

Synthesis: Methods B (48% yield, white solid). **^{1}H NMR** (400 MHz, DMSO-d_6): δ_H 12.23 (s, 1H, COOH), 7.99 (dd, J = 9.5 Hz, 1H, Ar-H), 7.62 (d, J = 8.8 Hz, 1H, Ar-H), 6.98 (d, J = 2.1 Hz, 1H, Ar-H), 6.94 (d, J = 8.6 Hz, 1H, Ar-H), 6.28 (d, J = 9.4 Hz, 1H, Ar-H), 4.09 (t, J = 6.5 Hz, 2H, CH_2), 2.39 (t, J = 7.3 Hz, 2H, CH_2), 1.96 (p, J = 6.9 Hz, 2H, CH_2). **^{13}C NMR** (100 MHz, DMSO): δ_C 174.0, 161.6, 160.3, 155.4, 144.3, 129.5, 112.7, 112.4, 112.3, 101.1, 67.4, 30.0, 24.0. **HRMS** (ESI) *m/z*: $[M-1H]^-$ calcd. for $C_{13}H_{12}O_5$ 247.0612; observed 247.0611.

4-(4-cyano-2,6-dimethylphenoxy)butanoic acid - (12)

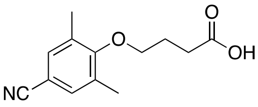

Synthesis: Methods A (90% yield, white solid). **^{1}H NMR** (400 MHz, DMSO-d_6): δ_H 12.16 (s, 1H, COOH), 7.53 (s, 2H, Ar-H), 3.82 (t, J = 6.4 Hz, 2H, CH_2), 2.55 (s, 6H, Ar-CH_3), 2.45 (t, J = 7.3 Hz, 2H, CH_2), 1.97 (p, J = 6.9 Hz, 2H, CH_2). **^{13}C NMR** (150 MHz, DMSO-d_6): δ_C 174.0, 159.5, 132.6, 132.4, 118.8, 106.1, 71.0, 30.0, 25.2, 15.6. LCMS (ESI) m/z: $[M-1H]^-$ calcd. for $C_{13}H_{12}NO_3$ 232.0979; observed 231.9.

4-(4-bromo-2,6-dimethylphenoxy)butanoic acid - (13)

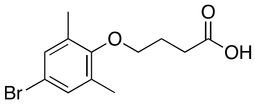

Synthesis: Methods A (94% yield, white solid). **^{1}H NMR** (400 MHz, DMSO-d_6): δ_H 12.14 (s, 1H, COOH), 7.21 (s, 2H, Ar-H), 3.73 (t, J = 6.3 Hz, 2H, CH_2), 2.45 (t, J =7.3 Hz, 2H, CH_2), 2.19 (s, 6H, ArCH_3), 1.95 (p, J = 6.9 Hz, 2H, CH_2).**^{13}C NMR** (100 MHz, $CDCl_3$): δ_C. 174.0, 154.6, 133.1, 130.8, 115.3, 70.6, 30.0, 25.1, 15.5. **HRMS** (ESI) *m/z*: $[M-1H]^-$ calcd. for $C_{12}H_{15}BrO_3$ 285.0132; observed 285.0130.

4-(4-bromo-3,5-dimethylphenoxy)butanoic acid - (14)

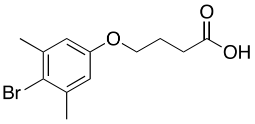

Synthesis: Methods B (69% yield, white solid). **^{1}H NMR** (400 MHz, $CDCl_3$): δ_H 6.63 (s, 2H, Ar-H), 3.98 (t, J = 6.1 Hz, 2H, CH_2), 2.57 (t, J = 7.3 Hz, 2H, CH_2), 2.37 (s, 6H, ArCH_3), 2.1 (tt, J = 7.3 Hz, 6.1 Hz, 2H, CH_2).**^{13}C NMR** (100 MHz, $CDCl_3$): δ_C 173.9, 157.0, 138.3, 117.1, 114.5, 66.5, 29.9, 24.0, 23.3. **HRMS** (ESI) *m/z*: $[M-1H]^-$ calcd. for $C_{12}H_{15}BrO_3$ 285.0132; observed 285.0135.

4-(4-cyclohexylphenoxy)butanoic acid (15)

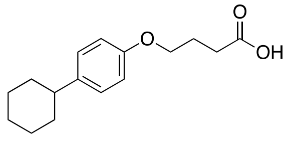

Synthesis: Method A (46%, white solid). **^{1}H NMR** (400 MHz, DMSO-d_6): δ_H 12.12 (s, 1H, COOH), 7.10 (d, J = 8.6 Hz, 2H, Ar-H), 6.82 (d, J = 8.6 Hz, 2H, Ar-H), 3.39 (t, J = 6.4 Hz, 2H, CH_2), 2.45–2.28 (m, 1H), 2.36 (t, J = 7.4 Hz, 2H, CH_2), 1.19 (p, J = 6.9 Hz, 2H, CH_2), 1.83–1.60

(m, 5H), 1.44–1.26 (m, 4H), 1.26–1.10 (m, 1H). **HRMS** (ESI) *m/z*: [M-1H]$^-$ calcd. for $C_{16}H_{22}O_3$ 261.1496; observed 261.1491.

4-(4-benzylphenoxy)butanoic acid - (16)

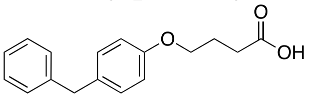

Synthesis: Methods A (87% yield, white solid). **^{1}H NMR** (400 MHz, DMSO-d_6): δ_H 12.14 (s, 1H, COOH), 7.43–7.24 (m, 2H, Ar-H), 7.24–7.03 (m, 5H, Ar-H), 6.84 (d, *J* = 8.5 Hz, 2H, Ar-H), 3.93 (t, *J* = 6.4 Hz, 2H, CH$_2$), 3.85 (s, 2H, CH$_2$), 2.37 (t, *J* =7.3 Hz, 2H, CH$_2$), 1.91 (p, *J* = 6.9 Hz, 2H, CH$_2$). **^{13}C NMR** (100 MHz, DMSO-d_6): δ_C 174.0, 156.7, 141.6, 133.1, 129.5, 128.4, 128.2, 125.7, 114.3, 66.4, 30.0, 24.2. **HRMS** (ESI) *m/z*: [M-1H]$^-$ calcd. for $C_{17}H_{18}O_3$ 269.1183; observed not detected.

4-(5-chloro-2-(2,4-dichlorophenoxy)phenoxy)butanoic acid - (17)

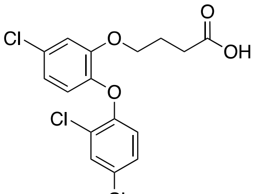

Synthesis: Methods A (54% yield, white solid). **^{1}H NMR** (400 MHz, DMSO-d_6): δ_H 12.08 (s, 1H, COOH), 7.68 (d, *J* = 2.5 Hz, 1H, Ar-H), 7.40–7.21 (m, 2H, Ar-H), 7.15 (d, *J* = 8.5 Hz, 1H, Ar-H), 7.04 (dd, *J* = 8.5 Hz, 2.5 Hz, 1H, Ar-H), 6.75 (d, *J* = 8.8 Hz, 1H, ArH), 3.98 (t, *J* = 6.2 Hz, 2H, CH$_2$), 2.08 (t, *J* = 7.3 Hz, 2H, CH$_2$), 1.72 (p, *J* = 6.9 Hz, 2H, CH$_2$). **^{13}C NMR** (100 MHz, DMSO-d_6): δ_C 174.1, 152.3, 150.7, 142.4, 130.0, 129.9, 128.5, 127.0, 123.3, 122.8, 121.1, 118.3, 115.0, 67.9, 29.6, 24.1. **HRMS** (ESI) *m/z*: [M-1H]$^-$ calcd. for $C_{16}H_{13}Cl_3O_4$ 372.9807; observed 372.9806.

4-(4-bromo-2,3-dimethylphenoxy)butanamide - (18)

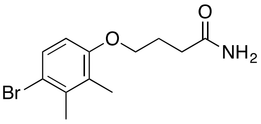

Synthesis: To a solution of the acid **H1** (60 mg, 0.2 mmol) and HATU (182 mg, 2.3 equiv) in THF (2 ml, 0.1 M of the acid) was added a solution of ammonium hydroxide 30% (0.180 ml, 8 equiv) The resulting yellow solution was stirred at room temperature for 16 h upon which additional amount of HATU (182 mg, 2.3 equiv) and a solution of ammonia in MeOH 7N (0.380 ml, 100 equiv) were added. Immediate white precipitate was formed, and the mixture was stirred for 3 h at room temperature. The mixture was then concentrated under vacuum and the crude was dissolved in MeOH and purified by preparative HPLC (A: 0.75% HCOOH in H_2O, B: 0.75% HCOOH in CH_3CN, 10→70% B over 15 min) to afford product of interest in 82% yield as a white solid. **^{1}H NMR** (400 MHz, DMSO-d_6): δ_H 7.34 (d, *J* = 8.8 Hz, 1H, Ar-H), 7.31 (bs, 1H, CONH$_2$), 6.76 (bs, 1H, CONH$_2$), 6.76 (d, *J* = 8.8 Hz, 1H, Ar-H), 3.92 (t, *J* = 6.3 Hz, 2H, CH$_2$), 2.29 (s, 3H, ArCH$_3$), 2.23 (t, *J* = 7.4 Hz, 2H, CH$_2$), 2.16 (s, 3H, ArCH$_3$), 1.92 (p, *J* = 6.9 Hz, 2H, CH$_2$). **^{13}C NMR** (100 MHz, DMSO-d_6): δ_C 173.6, 155.6, 136.1, 129.5, 126.7, 115.3, 111.1, 67.5, 31.4, 24.70, 19.5, 12.7. **HRMS** (ESI) *m/z*: [M-1H]$^-$ calcd. for $C_{12}H_{16}BrNO_2$ 284.0292; observed not detected.

4-(4-bromo-2,3-dimethylphenoxy)-*N*-methylbutanamide - (19)

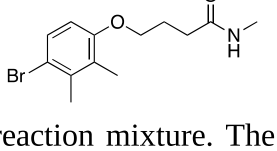

Synthesis: To a solution of acid (60 mg, 0.2 mmol) and HATU (182 mg, 2.3 equiv) in THF (2 ml, 0.1 M of the acid) was added a solution of methyl amine (0.835 ml, 2 M in THF, 8 equiv) The resulting yellow solution was stirred at room temperature for 16 h. Product was precipitated in the reaction mixture. The mixture was concentrated under vacuum and the crude was dissolved in MeOH and purified by preparative HPLC (A: 0.75% HCOOH in H_2O, B: 0.75% HCOOH in CH_3CN, 10→70% B over 15 min) 10 → 70% with to afford product of interest in 47% yield as a white solid. **^{1}H NMR** (400 MHz, DMSO-d_6): δ_H 7.76 (bs, 1H, CONH), 7.35 (d, *J* = 8.8 Hz, 1H, Ar-H), 6.75 (d, *J* = 8.8 Hz, 1H, Ar-H), 3.92 (t, *J* = 6.3 Hz, 2H, CH$_2$), 2.56 (d, *J* = 4.6 Hz, 3H, CONHCH$_3$), 2.29 (s, 3H, ArCH$_3$), 2.23 (t, *J* = 7.4 Hz, 2H, CH$_2$), 2.16 (s, 3H, ArCH$_3$), 1.93 (p, *J* = 6.9 Hz, 2H, CH$_2$). **^{13}C NMR** (100 MHz, DMSO-d_6): δ_C 172.3, 156.1, 136.6, 130.0, 127.2, 115.8, 111.6, 68.0, 32.2, 25.9, 25.3, 20.0, 13.1. **HRMS** (ESI) *m/z*: [M-1H]$^-$ calcd. for $C_{13}H_{18}BrNO_2$ 298.0448; observed not detected.

1-(4-(4-bromo-2,3-dimethylphenoxy)butanoyl)piperidine-3-carboxylic acid - (20)

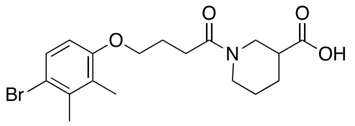

Synthesis: Method C (56% yield, white solid). Note that the amide bond for this compound isomerizes (*E*/*Z*) at room temperature, which was observed in the following recorded NMR data. The NMR signals are then reported as such. **^{1}H NMR** (400 MHz, DMSO-d_6): δ_H 12.45 (s, 1H, COOH), 7.35 (d, *J* = 8.8 Hz, 1H, Ar-H), 6.77 (d, *J* = 8.8 Hz, 1H, Ar-H), 4.44 (t, *J* = 6.1 Hz, 1/2H), 3.96 (t, *J* = 6.1 Hz, 2H, CH_2), 3.86–3.60 (m, 3/2H), 3.45–3.19 (m, 3H, CH_2), 3.09–2.94 (m, 1H, CH_2), 2.79–2.68 (m, 1/2H, CH_2), 2.59–2.38 (m, 14H, CH_2), 2.30 (s, 3H, $ArCH_3$), 2.16 (s, 3H, $ArCH_3$), 2.04–1.82 (m, 3H, CH_2), 1.79–1.46 (m, 2H, CH_2), 1.46–1.28 (m, 1H, CH_2). **^{13}C NMR** (100 MHz, DMSO-d_6): δ_C 174.8, 174.8, 170.4, 170.4, 156.1, 136.6, 130.0, 127.2, 115.7, 111.6, 67.9, 67.8, 47.1, 45.6, 43.5, 41.7, 41.1, 29.2, 27.2, 25.0, 24.0, 20.0. **HRMS** (ESI) *m/z*: $[M-1H]^-$ calcd. for $C_{18}H_{24}BrNO_4$ 396.0816; observed 396.0825.

2-(4-(4-bromo-2,3-dimethylphenoxy)butanamido)-2-phenylacetic acid - (21)

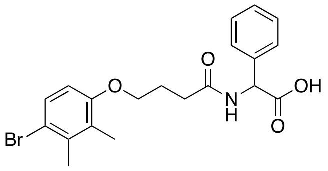

Synthesis: Method C (58% yield, white solid). **^{1}H NMR** (400 MHz, DMSO-d_6): δ_H 12.81 (s, 1H, COOH), 8.63 (d, *J* = 7.5 Hz, 1H, CONH), 7.45–7.22 (m, 6H, ArH), 6.73 (d, *J* = 8.8 Hz, 1H, Ar-H), 5.33 (d, *J* = 7.5 Hz, 1H, CONHCH), 3.96 (t, *J* = 6.4 Hz, 2H, CH_2), 2.38 (td, *J* = 7.0 Hz, 2.2 Hz, 2H, CH_2), 2.29 (s, 3H, $ArCH_3$), 2.15 (s, 3H, $ArCH_3$), 1.95 (p, *J* = 6.9 Hz, 2H, CH_2). **^{13}C NMR** (100 MHz, DMSO-d_6): δ_C 172.4, 172.0, 156.1, 137.7, 136.6, 130.0, 128.9, 128.3, 127.2, 115.8, 111.6, 67.9, 56.7, 31.8, 25.3, 20.0, 13.2. **HRMS** (ESI) *m/z*: $[M-1H]^-$ calcd. for $C_{20}H_{22}BrNO_4$ 418.0659; observed 418.0671.

1-(4-(4-bromo-2,3-dimethylphenoxy)butanoyl)piperidine-2-carboxylic acid - (22)

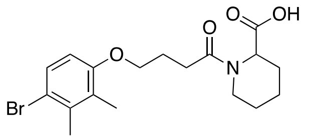

Synthesis: Method C (65% yield, white solid). Note that the amide bond for this compound isomerizes (*E*/*Z*) at room temperature, which was observed in the following recorded NMR data at room temp. The NMR signals are then reported as such.**^{1}H NMR** (400 MHz, DMSO-d_6): δ_H 12.84 (s, 1H, COOH), 7.36 (dd, *J* = 8.8 Hz, 2.2 Hz, 1H, Ar-H), 6.77 (dd, *J* = 8.8 Hz, 2.2 Hz, 1H, Ar-H), 5.08 (dd, *J* = 5.7 Hz, 1.6 Hz, 2/3H, NCHCOOH, *E/Z*-amide), 4.76–4.67 (m, 1/3H, NCHCOOH, *E/Z*-amide), 4.34 (d, *J* = 13.2 Hz, 1/3H, NCH_2, *E/Z*-amide), 4.03–3.90 (m, 2H, CH_2), 3.80 (d, *J* = 13.2 Hz, 2/3H, NCH_2, *E/Z*-amide), 3.12 (td, *J* = 13.1 Hz, 2.9 Hz, 2/3H, NCH_2, *E/Z*-amide), 2.63–2.41 (m, interfere with DMSO signal), 2.37–2.25 (m, 2/3H, NCH_2), 2.30 (s, 3H, $ArCH_3$), 2.17 (s, 3H, $ArCH_3$), 2.21–2.05 (m, 2H, CH_2), 1.95 (p, *J* = 6.9 Hz, 2H, CH_2), 1.72–1.43 (m, 3H, CH_2), 1.43–1.05 (m, 2H, CH_2). **^{13}C NMR** (100 MHz, DMSO-d_6): δ_C172.3, 171.4, 171.3, 155.4, 135.9, 135.8, 129.3, 126.5, 115.1, 110.9, 67.14, 67.10, 55.1, 51.0, 42.4, 28.6, 26.0, 24.6, 24.30, 20.4, 19.3, 12.5, 12.4. **HRMS** (ESI) *m/z*: $[M-1H]^-$ calcd. for $C_{18}H_{24}BrNO_4$ 396.0816; observed 396.0827.

1-(4-(4-bromo-2,3-dimethylphenoxy)butanoyl)piperidine-4-carboxylic acid - (23)

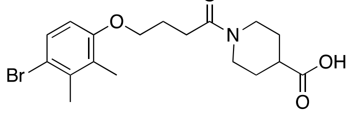

Synthesis: Method C as above (40% yield, white solid). **^{1}H NMR** (400 MHz, DMSO-d_6): δ_H 12.34 (s, 1H, COOH), 7.35 (d, *J* = 8.8 Hz, 1H, Ar-H), 6.77 (d, *J* = 8.8 Hz, 1H, Ar-H), 4.22 (dt, *J* = 13.0 Hz, 3.9 Hz, 1H, NCHCOOH), 3.96 (t, *J* = 6.4 Hz, 2H, CH_2) 3.8 (dt, *J* = 13.0 Hz, 3.9 Hz, 1H, NCH), 3.07 (ddd, *J* = 13.5 Hz, 11.4 Hz, 2.2 Hz, 1H), 2.71 (ddd, *J* = 13.5 Hz, 11.4 Hz, 2.2 Hz, 1H), 2.54–2.42 (m, interfere with DMSO signal), 2.30 (s, 3H, $ArCH_3$), 2.15 (s, 3H, $ArCH_3$), 1.95 (p, *J* = 6.9 Hz, 2H, CH_2), 1.87–1.72 (m, 3H, CH_2), 1.59–1.19 (m, 2H, CH_2). **^{13}C NMR** (100 MHz, DMSO-d_6): δ_C 176.0, 170.3, 156.1, 136.6, 130.0, 127.2, 115.7, 111.6, 67.9, 44.6, 29.1, 28.8, 28.1, 25.0, 20.0, 13.2. **HRMS** (ESI) *m/z*: $[M-1H]^-$ calcd. for $C_{18}H_{24}BrNO_4$ 396.0816; observed 396.0825.

2-(4-(4-bromo-2,3-dimethylphenoxy)butanamido)-4,5-difluorobenzoic acid - (24)

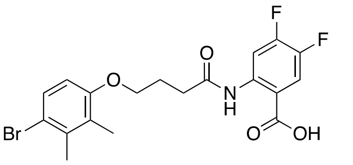

Synthesis: Method C (56% yield, white solid). **^{1}H NMR** (400 MHz, DMSO-d_6): δ_H 14.01 (bs, 1H), 11.22 (s, 1H), 8.53 (dd, J = 13.8 Hz, 7.5 Hz, 1H, ArH), 7.92 (dd, J = 11.3 Hz, 9.1 Hz, 1H, ArH), 7.33 (d, J = 8.8 Hz, 1H, ArH), 6.76 (d, J = 8.8 Hz, 1H, ArH), 4.0 (t, J = 6.2 Hz, 2H, CH$_2$), 2.6 (t, J = 7.3 Hz, 2H, CH$_2$), 2.26 (s, 3H, ArCH$_3$), 2.11 (s, 3H, ArCH$_3$), 2.14–2.03 (m, 2H, CH$_2$). **^{13}C NMR** (100 MHz, DMSO-d_6): δ_C 170.9, 167.5, 155.2, 151.8 (dd, $^{1,2}J_{CF}$ = 250 Hz, 11 Hz), 143.7 (dd, $^{1,2}J_{CF}$ = 243 Hz, 14 Hz), 138.2 (d, $^3J_{CF}$ = 11 Hz), 135.8, 129.2, 126.4, 119.2 (d, $^2J_{CF}$ = 18 Hz), 115.1, 113.0, 110.8, 108.4 (d, $^2J_{CF}$ = 23 Hz), 66.8, 33.7, 24.1, 19.2, 12.3. **HRMS** (ESI) m/z: [M-1H]$^-$ calcd. for $C_{19}H_{18}BrF_2NO_4$ 440.0315; observed 440.0307.

4-(4-bromo-2,3-dimethylphenoxy)-N-((trifluoromethyl)sulfonyl)butanamide (25)

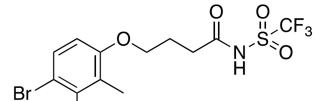

Synthesis: To a solution of acid derivative **H1** (30 mg, 0.1 mmol) and HATU (99 mg, 0.26 mmol, 2.5 equiv. to the acid) in DMF (0.5 ml, 0.2 M of the acid) was added DIEPA (36 µl, 2 equiv.), and the mixture was stirred for 30 min. In another vial, sulfonamide (77 mg, 5 equiv.) and NaH 60% dispersion in mineral oil (4 equiv.) was mixed at 0 °C in DMF (2.3 ml, 0.2 M of sulfonamide) and stirred for 1 h. The acid solution was then transferred to the sulfonamide solution dropwise at 0 °C and the resulting mixture was stirred for 18 h at room temperature. The reaction was monitored with LCMS. The mixture was then diluted with EtOAc and washed with HCl (1 M) solution, H_2O and brine. The organic layer was dried over Na_2SO_4 and concentrated under vacuum. The crude product was dissolved in DMSO and purified by HPLC (A: 0.75% HCOOH in H_2O, B: 0.75% HCOOH in CH_3CN, 20→100% B over 15 min) to afford the product of interest. (52% yield, amorphous solid). **^{1}H NMR** (400 MHz, DMSO-d_6): δ_H 7.34 (d, J = 8.8 Hz, 1H, Ar-H), 6.76 (d, J = 8.8 Hz, 1H, Ar-H), 3.93 (t, J = 6.4 Hz, 2H, CH$_2$), 2.38 (t, J = 7.3 Hz, 2H, CH$_2$), 2.29 (s, 3H, ArCH$_3$), 2.15 (s, 3H, ArCH$_3$), 1.91 (p, J = 6.9 Hz, 2H, CH$_2$). **^{13}C NMR** (100 MHz, DMSO-d_6): δ_C 174.6, 156.1, 136.6, 130.0, 127.2, 120.0 (q, J = 315 Hz), 115.8, 111.6, 67.6, 34.6, 24.7, 20.0, 13.1. **HRMS** (ESI) m/z: [M-1H]$^-$ calcd. for $C_{13}H_{15}BrF_3NO_4S$ 415.9784; observed 415.9796.

4-(4-bromo-2,3-dimethylphenoxy)-N-(methylsulfonyl)butanamide (26)

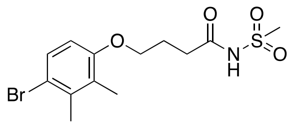

Synthesis: As for compound **25** (49% yield, white solid). **^{1}H NMR** (400 MHz, DMSO-d_6): δ_H 11.74 (s, 1H, SO$_2$NHCO), 7.35 (d, J = 8.8 Hz, 1H, Ar-H), 6.76 (d, J = 8.8 Hz, 1H, Ar-H), 3.94 (t, J = 6.2 Hz, 2H, CH$_2$), 3.21 (s, 3H, SO$_2$CH$_3$), 2.47 (t, J = 7.5 Hz, 2H, CH$_2$), 2.30 (s, 3H, ArCH$_3$), 2.16 (s, 3H, ArCH$_3$), 1.96 (p, J = 6.9 Hz, 2H, CH$_2$). **^{13}C NMR** (100 MHz, DMSO-d_6): δ_C 172.5, 155.8, 136.4, 129.8, 127.0, 115.7, 111.4, 66.9, 40.9, 32.3, 23.9, 19.5, 13.0. **HRMS** (ESI) m/z: [M-1H]$^-$ calcd. for $C_{13}H_{18}BrNO_4S$ 362.0067; observed 362.0078.

4-(4-bromo-2,3-dimethylphenoxy)-N-(phenylsulfonyl)butanamide (27)

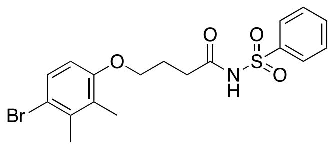

Synthesis: As for compound **25** (57% yield, white solid). **^{1}H NMR** (400 MHz, DMSO-d_6): δ_H 12.17 (s, 1H, SO$_2$NHCO), 7.9 (dd, J = 8.3 Hz, 1.5 Hz, 2H, Ar-H), 7.69 (t, J = 7.4 Hz, 1H, Ar-H), 7.59 (t, J = 7.8 Hz, 2H, Ar-H), 7.33 (d, J = 8.8 Hz, 1H, Ar-H), 6.76 (d, J = 8.8 Hz, 1H, Ar-H), 3.82 (t, J = 6.2 Hz, 2H, CH$_2$), 2.41 (t, J = 7.5 Hz, 2H, CH$_2$), 2.29 (s, 3H, ArCH$_3$), 2.10 (s, 3H, ArCH$_3$), 1.96 (p, J = 6.9 Hz, 2H, CH$_2$). **^{13}C NMR** (100 MHz, DMSO-d_6): δ_C171.4, 155.5, 139.1, 136.2, 133.5, 129.5, 129.1, 127.4, 126.8, 115.4, 111.1, 66.9, 32.0, 23.6, 19.6, 12.7. **HRMS** (ESI) m/z: [M-1H]$^-$ calcd. for $C_{18}H_{20}BrNO_4S$ 424.0224; observed 424.0237.

4-(4-bromo-2,3-dimethylphenoxy)butan-1-ol (28)

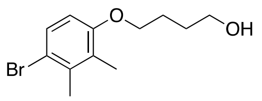

Synthesis: Acid derivative **H1** (52 mg, 0.18 mmol) was dissolved in anhydrous THF (0.5 ml) under N_2. The solution was cooled to 0 °C, and $BH_3{\cdot}(CH_3)_2S$ (1.1 equiv., 99 µl) was added dropwise. The reaction mixture was stirred for 18 h at room temperature. Then solvents were evaporated, and the residue was dissolved in EtOAc, washed twice with saturated $NaHCO_3$, dried over Na_2SO_4 and concentrated under vacuum. The crude was loaded to silica gel and purified by chromatography (EtOAc/Heptane, 1:1) linear gradient and a product of R_f = 0.27 (EtOAc/Heptane, 1:1) was isolated to afford arylbutan-1-ol as colorless oil (75% yield, colorless oil). **^{1}H NMR** (400 MHz, DMSO-*d*$_6$): δ_H 7.35 (d, *J* = 8.8 Hz, 1H, Ar-H), 6.76 (d, *J* = 8.8 Hz, 1H, Ar-H), 4.44 (t, *J* = 5.2 Hz, 1H, OH), 3.92 (t, *J* = 6.4 Hz, 2H, CH_2), 3.45 (td, *J* = 6.4 Hz, 5.2 Hz, 2H, CH_2), 2.29 (s, 3H, $ArCH_3$), 2.16 (s, 3H, $ArCH_3$), 1.81–1.69 (m, 2H, CH_2), 1.63–1.51 (m, 2H, CH_2). **^{13}C NMR** (100 MHz, DMSO-*d*$_6$): δ_C 155.7, 136.1, 129.5, 126.7, 115.2, 111.1, 67.9, 60.4, 29.0, 25.5, 19.5, 12.7. **HRMS** (ESI) *m/z*: $[M-1H]^-$ calcd. for $C_{12}H_{17}BrO_2$ 271.0339; observed not detected.

Methyl 4-(4-bromo-2,3-dimethylphenoxy)butanoate (29)

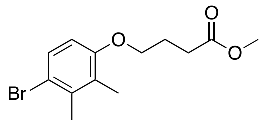

Synthesis: To a solution of acid **H1** (60 mg, 0.2 mmol) in CH_2Cl_2/MeOH (9:1) (2 ml, 0.1M of acid) was added 2,6-lutidine (26 µl, 1.1 equiv) and stirred until a clear solution was developed (ca. 5 min). TMS-diazomethane (2M in hexane, 115 µl, 0.22 mmol) was added dropwise at 0 °C, and the reaction mixture was stirred at room temperature for 1 h. The mixture was diluted with CH_2Cl_2 and washed with H_2O, dried over Na_2SO_4, concentrated under vacuum and loaded to silica gel and purified by chromatography using EtOAc/Heptane 10→40% and a product of ca. R_f = 0.5 (EtOAc/Heptane 1:3) was isolated to afford the corresponding ester in 79% yield, as a colorless oil. **^{1}H NMR** (400 MHz, DMSO-*d*$_6$): δ_H 7.36 (d, *J* = 8.8 Hz, 1H, Ar-H), 6.76 (d, *J* = 8.8 Hz, 1H, Ar-H), 3.96 (t, *J* = 6.2 Hz, 2H, CH_2), 3.60 (s, 3H, CO_2CH_3), 2.54–2.48 (m, 2H, CH_2, overlapped with DMSO signal), 2.30 (s, 3H, $ArCH_3$), 2.14 (s, 3H, $ArCH_3$), 1.99 (p, *J* = 6.8 Hz, 2H, CH_2). **^{13}C NMR** (100 MHz, DMSO-*d*$_6$): δ_C 172.8, 155.4, 136.0, 129.4, 126.2, 115.2, 110.9, 66.9, 51.1, 29.9, 24.1, 19.4, 12.5. **HRMS** (ESI) *m/z*: $[M+H]^+$ calcd. for $C_{13}H_{17}BrO_3$ 301.0434; observed not detected.

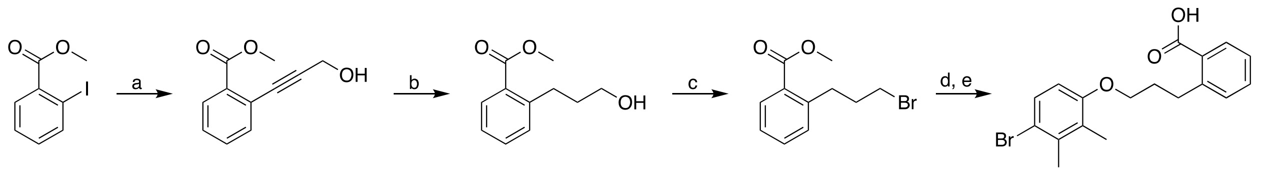

30

Synthesis:

Step a: Phenyl iodide derivative (850 mg, 3.2 mmol), $PdCl_2(PPh_3)_2$ (6 mol %) and CuI (3 mol %) was dissolved in anhydrous and degassed THF/TEA (1:3, 21 ml, 0.15 M). The mixture was degassed further by bubbling N_2 and propargyl alcohol (3.0 equiv., 409 µl) was added under N_2 atmosphere. The mixture was stirred at 40 °C in microwave reactor for 30 min upon which the reaction was completed according to TLC (EtOAc/Heptane 1:3, R_f = 0.1). The reaction mixture was diluted with EtOAc/MeOH 10%, filtered over a pad of Celite and concentrated under vacuum. The crude was loaded to silica gel and purified by chromatography using EtOAc/Heptane 10→33% linear gradient and product was isolated as yellow oil in quantitative yield. **^{1}H NMR** (400 MHz, $CDCl_3$): δ_H 7.92 (dd, *J* = 7.7 Hz, 1.5 Hz, 1H, Ar-H), 7.53 (dd, *J* = 7.7 Hz, 1.5 Hz, 1H, Ar-H), 7.45 (td, *J* = 7.7 Hz, 1.5 Hz, 1H, Ar-H), 7.35 (td, *J* = 7.7 Hz, 1.5 Hz, 1H, Ar-H), 4.53 (d, *J* = 6.2 Hz, 2H, CH_2), 3.9 (s, 3H, CO_2CH_3), 2.08 (t, *J* = 6.2 H, 1H, OH).

Step b: To a solution of the alkyne (300 mg, 1.577 mmol) in EtOAc/MeOH (1:1, 3 ml, 0.5 M) was added Pd/10% on C (20 mol%), and the mixture was stirred under H_2 atmosphere for 2 h at room temperature. The reaction was monitored with ^{1}H NMR (A sample was filtered, concentrated and dissolved in $CDCl_3$), which showed full conversion. The reaction mixture was filtered over a pad of Celite and concentrated under vacuum to afford product in 59% yield and ca. 90% purity according to NMR. The product was used for the next step without further purification. **^{1}H NMR** (400 MHz, $CDCl_3$): δ_H 7.88 (dd, J = 7.7 Hz, 1.5 Hz, 1H, Ar-H), 7.44 (td, J = 7.7 Hz, 1.5 Hz, 1H, Ar-H), 7.33–7.19 (m, 2H, Ar-H), 3.9 (s, 3H, CO_2CH_3), 3.62 (t, J = 6.0 H, 2H, CH_2), 3.06 (t, J = 7.4 H, 2H, CH_2), 1.91 (tt, J = 7.4 H, 6.0 Hz, 2H, CH_2).

Step c: To a solution of alcoholic compound (130 mg, 0.67 mmol) in anhydrous CH_2Cl_2 (1.3 ml, 0.5 M) under N_2 was added triphenylphosphine (1.2 equiv., 210 mg) followed by carbon tetrabromide (266 mg, 1.2 equiv.) slowly at 0 °C. The resulting mixture was stirred for 30 min and the reaction was monitored with TLC (EtOAc/Heptane 1:3), which indicated full conversion. The crude was loaded on silica gel and purified by chromatography using EtOAc/Heptane 1→12 linear gradient and the product was isolated in 94% yield. **^{1}H NMR** (400 MHz, $CDCl_3$): δ_H 7.93 (dd, J = 7.7 Hz, 1.5 Hz, 1H, Ar-H), 7.46 (td, J = 7.7 Hz, 1.5 Hz, 1H, Ar-H), 7.35–7.25 (m, 2H, Ar-H), 3.91 (s, 3H, CO_2CH_3), 3.47 (t, J = 6.0 H, 2H, CH_2), 3.13 (t, J = 7.4 H, 2H, CH_2), 2.21 (p, J = 7.2 H, 2H, CH_2).

2-(3-(4-bromo-2,3-dimethylphenoxy)propyl)benzoic acid (30)

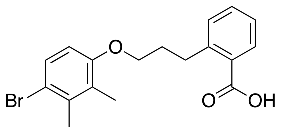

Step d and e: Method A (85% yield, white solid). **^{1}H NMR** (400 MHz, DMSO-d_6): δ_H 12.87 (s, 1H, COOH), 7.79 (dd, J = 7.6 Hz, 1.0 Hz, 1H, Ar-H), 7.46 (dd, J = 7.6 Hz, 1.1 Hz, 1H, Ar-H), 7.40–7.24 (m, 3H), 6.74 (d, J = 8.9 Hz, 1H, Ar-H), 3.93 (t, J = 6.2 Hz, 2H, CH_2), 3.09 (t, J = 7.6 Hz, 2H, CH_2), 2.30 (s, 3H, $ArCH_3$), 2.17 (s, 3H, $ArCH_3$), 2.05–1.93 (m, 2H, CH_2). **^{13}C NMR** (100 MHz, DMSO-d_6): δ_C 169.3, 156.2, 142.9, 136.6, 132.1, 131.2, 131.1, 130.7, 130.7, 127.2, 126.5, 115.7, 111.4, 67.9, 31.2, 30.6, 20.0, 13.1. **HRMS** (ESI) m/z: $[M-1H]^-$ calcd. for $C_{18}H_{19}BrO_3$ 361.0445; observed 361.0461

5-(4-bromo-2,3-dimethylphenoxy)pentanoic acid - (31)

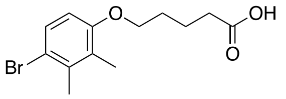

Synthesis: Methods A (86% yield, white solid, purified by preparative HPLC). **^{1}H NMR** (400 MHz, DMSO-d_6): δ_H 12.06 (s, 1H, COOH), 7.36 (d, J = 8.9 Hz, 1H, Ar-H), 6.77 (d, J = 8.8 Hz, 1H, Ar-H), 3.94 (t, J = 6.0 Hz, 2H), 2.33–2.26 (m, 2H), 2.30 (s, 3H, $ArCH_3$), 2.15 (s, 3H, $ArCH_3$), 1.63–1.56 (m, 4H). **^{13}C NMR** (150 MHz, DMSO-d_6): δ_C 174.8, 156.2, 136.6, 130.0, 127.2, 115.7, 111.6, 68.1, 33.7, 28.6, 21.7, 20.0, 13.1. **HRMS** (ESI) m/z: $[M-1H]^-$ calcd. for $C_{13}H_{17}BrO_3$ 299.0288; observed not detected.

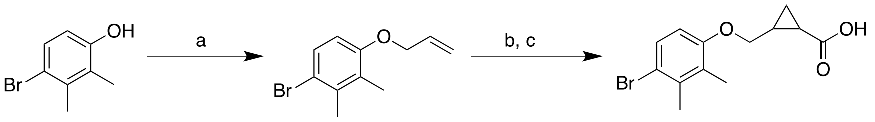

Synthesis:

Step a: Williamson ether synthesis as described in Method A (88% yield, colorless oil). **^{1}H NMR** (400 MHz, DMSO-d_6): δ_H 7.32 (d, J = 8.8 Hz, 1H, Ar-H), 6.59 (d, J = 8.8 Hz, 1H, Ar-H), 6.05 (ddt, J = 17.3 Hz, 10.6 Hz, 5.1 Hz, 1H, =CH), 5.41 (dq, J = 17.3 Hz, 1.7 Hz, 1H, =CH_2), 5.28 (dq, J = 17.3 Hz, 1.6 Hz, 1H, =CH_2), 4.5 (dt, J = 5.1 Hz, 1.6 Hz, 1H, CH_2), 2.37 (s, 3H, $ArCH_3$), 2.24 (s, 3H, $ArCH_3$).

Step b: 1-(Allyloxy)-4-bromo-2,3-dimethylbenzene (156 mg, 0.65 mmol, 1.2 equiv.) and dirhodium tetraacetate (1 mol%) was dissolved in CH_2Cl_2 (1.3 ml, 0.5 M of the alkene). A solution of ethyl diazoacetate (57 µl, 0.54 mmol, 1 equiv.) in CH_2Cl_2 (0.5 ml) was added *via* a syringe pump over a period of approximately 5 h under N_2 atmosphere at room temperature and the mixture was stirred for additional 2 h. Note that the cylcoproponation products were obtained as a racemic *E* and *Z* diastereomeric mixture. The crude was then loaded on silica gel and purified by chromatography using EtOAc in petroleum ether (bp 40–60), linear gradient 0→5% and 25 g SNAP ULTRA cartridge with flow rate of 45 ml/min. This allowed the separation of the *E* and *Z* diastereomers in their racemic form with 68% overall yield and ca. 2:1 ratio as colorless oils. Note that these compounds exhibit a slightly low absorption at 254 nm and it was important to visualize the TLC with Seebach's staining solution to clearly differentiate the spots.

Step c: The *E* or *Z* cyclopropyl ethyl ester obtained above was dissolved in THF/MeOH/H_2O (3:1:1, 0.1 M) in a microwave vial and LiOH.H_2O (5 equiv.) was added. The mixture was heated in a microwave reactor for 15 min at 65 °C. TLC showed full conversion (EtOAc/Heptane 1:1, stained with bromocresol green). Volatile solvents were evaporated under vacuum and the remaining aqueous layer was acidified with HCl (6 M) to pH < 3 and the crude was dissolved in DMSO and purified on preparative HPLC (A: 0.75% HCOOH in H_2O, B: 0.75% HCOOH in CH_3CN, 20→100% B over 25 min) to afford the product of interest (27% yield for **32** and 78% yield **33**).

(1*S*,2*S*)- and (1*R*,2*R*)-2-((4-bromo-2,3-dimethylphenoxy)methyl)cyclopropane-1-carboxylic acid - (32)

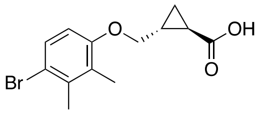

^{1}H NMR (600 MHz, DMSO-d_6): δ_H 12.18 (s, 1H, COO<u>H</u>), 7.36 (d, *J* = 8.8 Hz, 1H, Ar-<u>H</u>), 6.76 (d, *J* = 8.8 Hz, 1H, Ar-<u>H</u>), 4.38–4.25 (m, 1H, OC<u>H</u>$_2$), 3.93–3.82 (m, 1H, OC<u>H</u>$_2$), 2.30 (s, 3H, ArC<u>H</u>$_3$), 2.15 (s, 3H, ArC<u>H</u>$_3$), 1.86–1.69 (m, 2H, C<u>H</u>, C<u>H</u>), 1.13 (ddd, *J* = 8.1 Hz, 8.4 Hz, 4.4 Hz, 1H, C<u>H</u>$_2$), 0.99–0.93 (m, 1H, C<u>H</u>$_2$). **^{13}C NMR** (150 MHz, DMSO-d_6): δ_C 173.8, 156.2, 136.5, 130.0, 127.4, 115.8, 111.9, 67.0, 20.0, 19.9, 17.5, 13.2, 11.9. **HRMS** (ESI) *m/z*: $[M-1H]^-$ calcd. for $C_{13}H_{15}BrO_3$ 297.0132; observed 297.0137.

(1*R*,2*S*)- and (1*S*,2*R*)-2-((4-bromo-2,3-dimethylphenoxy)methyl)cyclopropane-1-carboxylic acid - (33)

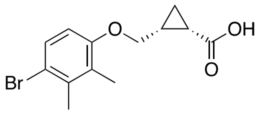

^{1}H NMR (600 MHz, DMSO-d_6): δ_H 12.22 (s, 1H, COO<u>H</u>), 7.36 (d, *J* = 8.7 Hz, 1H, Ar-<u>H</u>), 6.76 (d, *J* = 8.7 Hz, 1H, Ar-<u>H</u>), 3.98 (dd, *J* = 10.6 Hz, 6.0 Hz, 1H, OC<u>H</u>$_2$), 3.64 (dd, *J* = 10.6 Hz, 6.0 Hz, 1H, OC<u>H</u>$_2$), 2.30 (s, 3H, ArC<u>H</u>$_3$), 2.17 (s, 3H, ArC<u>H</u>$_3$), 1.77–1.64 (m, 1H, C<u>H</u>), 1.61 (ddd, *J* = 4.3 Hz, 8.7 Hz, 4.2 Hz, 1H, C<u>H</u>), 1.08 (ddd, *J* = 4.6 Hz, 4.3 Hz, 8.5 Hz, 1H, C<u>H</u>$_2$), 0.97 (ddd, *J* = 8.3 Hz, 5.9 Hz, 4.2 Hz, 1H, C<u>H</u>$_2$). **^{13}C NMR** (150 MHz, DMSO-d_6): δ_C 174.3, 155.4, 136.2, 129.5, 126.9, 115.6, 111.7, 69.7, 20.5, 19.5, 18.0, 12.7, 12.1. **HRMS** (ESI) *m/z*: $[M-1H]^-$ calcd. for $C_{13}H_{15}BrO_3$ 297.0132; observed 297.0131.
